# Effective strategies for Fecal Immunochemical Tests (FIT) programs to improve colorectal cancer screening uptake among populations with limited access to the healthcare system: a rapid review

**DOI:** 10.1186/s12913-024-10573-4

**Published:** 2024-01-23

**Authors:** Ana Paula Belon, Emily McKenzie, Gary Teare, Candace I. J. Nykiforuk, Laura Nieuwendyk, Minji (Olivia) Kim, Bernice Lee, Kamala Adhikari

**Affiliations:** 1 Centre for Healthy Communities, School of Public Health, University of Alberta, Edmonton, Canada; 2 Provincial Population and Public Health, Alberta Health Services, Calgary, Canada; 3 Department of Community Health Sciences, Cummings School of Medicine, University of Calgary, Calgary, Canada; 4 Health Evidence and Impact, Alberta Health Services, Calgary, Canada

**Keywords:** Fecal immunochemical test (FIT), Colorectal cancer, Cancer screening, Cancer prevention, Public health, Health equity, Rapid review

## Abstract

**Background:**

Colorectal cancer (CRC) is one of the leading causes of cancer death globally. CRC screening can reduce the incidence and mortality of CRC. However, socially disadvantaged groups may disproportionately benefit less from screening programs due to their limited access to healthcare. This poor access to healthcare services is further aggravated by intersecting, cumulative social factors associated with their sociocultural background and living conditions. This rapid review systematically reviewed and synthesized evidence on the effectiveness of Fecal Immunochemical Test (FIT) programs in increasing CRC screening in populations who do not have a regular healthcare provider or who have limited healthcare system access.

**Methods:**

We used three databases: Ovid MEDLINE, Embase, and EBSCOhost CINAHL. We searched for systematic reviews, meta-analysis, and quantitative and mixed-methods studies focusing on effectiveness of FIT programs (request or receipt of FIT kit, completion rates of FIT screening, and participation rates in follow-up colonoscopy after FIT positive results). For evidence synthesis, deductive and inductive thematic analysis was conducted. The findings were also classified using the Cochrane Methods Equity PROGRESS-PLUS framework. The quality of the included studies was assessed.

**Results:**

Findings from the 25 included primary studies were organized into three intervention design-focused themes. Delivery of culturally-tailored programs (e.g., use of language and interpretive services) were effective in increasing CRC screening. Regarding the method of delivery for FIT, specific strategies combined with mail-out programs (e.g., motivational screening letter) or in-person delivery (e.g., demonstration of FIT specimen collection procedure) enhanced the success of FIT programs. The follow-up reminder theme (e.g., spaced out and live reminders) were generally effective. Additionally, we found evidence of the social determinants of health affecting FIT uptake (e.g., place of residence, race/ethnicity/culture/language, gender and/or sex).

**Conclusions:**

Findings from this rapid review suggest multicomponent interventions combined with tailored strategies addressing the diverse, unique needs and priorities of the population with no regular healthcare provider or limited access to the healthcare system may be more effective in increasing FIT screening. Decision-makers and practitioners should consider equity and social factors when developing resources and coordinating efforts in the delivery and implementation of FIT screening strategies.

**Supplementary Information:**

The online version contains supplementary material available at 10.1186/s12913-024-10573-4.

## Background

Colorectal cancer (CRC) is the third most diagnosed cancer and the second most common cause of cancer death worldwide [1, 2]. Screening of CRC is highly effective at reducing the incidence and mortality of CRC, through the early detection of pre-cancerous polyps or CRC cases as well as facilitating early management and treatment [1, 3–5]. If detected early, more than 90% of cases can be successfully treated and the significant risk of CRC-associated mortality can be reduced, with patients surviving at least five years [1, 3, 4]. Subsequently, the total healthcare cost for managing CRC can be decreased significantly [5].

Endoscopy-based (colonoscopy or sigmoidoscopy) and stool-based (Fecal Occult Blood Test (FOBT): Fecal Immunochemical Test (FIT) or Guaiac Fecal Occult Blood Test (gFOBT)) tests are the most used effective screening modalities for the early detection of CRC [1, 6]. Program guideline recommendations for CRC screening, including target age groups and the choice of screening modalities, vary by country [1]. The FIT kits have been recommended for population-based programs [1]. High completion to FIT screening is essential for achieving benefits; however, screening rates remain suboptimal even in the high-income countries with established cancer screening programs [7, 8].

Health system access factors including not having a regular healthcare provider (i.e., with whom the patient develops a long-term relationship for assessment of physical and mental health issues) and fewer visits with general practitioner (GPs) are associated with lower rates of CRC screening [8–10]. Socially disadvantaged groups benefit less from screening programs because of their multiple, cumulative intersecting vulnerabilities that may lead to limited access to the healthcare system [9]. Uptake varies considerably by sociodemographic factors including ethnicity, educational attainment, language spoken, area of residence, income, and marital status [11–15]. For instance, rural residents are less likely to have regular screening as they are less likely to have a regular GP [15, 16], make fewer visits to GPs [17], and need to travel further to seek care [18].

Systematic reviews [19–21] indicate that multicomponent interventions are most effective in increasing CRC screening uptake among patients, and this is corroborated by the Community Preventive Service Task Force– a non-federal panel of experts created by the United States Department of Health and Human Services to guide population health strategies [22–24]. The multicomponent intervention approach combines two or more patient-centered interventions targeted at multiple levels (patients, providers and organizational or healthcare systems) to increase community demand and access, while providing screening services to promote CRC screening uptake among patients [23, 24]. Interventions that include patient reminders, patient education, and improved FIT kit access help address factors contributing to low CRC screening rates [22–24].

However, the effectiveness of multicomponent interventions that are targeted to address the lower CRC screening participation rates of disadvantaged populations with limited access to the healthcare system has not been reviewed or synthesized. The synthesized effectiveness evidence is critical for guiding the future design and implementation of population-wide FIT programs tailored to these disadvantaged groups. This rapid review aimed to systematically review and synthesize evidence on the effectiveness of FIT programs to increase CRC screening in populations who do not have a regular healthcare provider or are considered disadvantaged regarding healthcare system access (e.g., immigrants, low-income populations).

## Methods

This work was developed for a provincial healthcare authority (Alberta Health Services) in the Canadian province of Alberta. The purpose was to inform time-sensitive decisions in the provincial health system for increased effectiveness of FIT programs for population groups with limited healthcare access. Therefore, the rapid review method was deemed to be the most suitable for this work to streamline the rigorous process of systematically reviewing the literature. Due to its faster nature, the rapid review facilitates the partnership between researchers and decision-makers and ensures meaningful, continuing engagement of the latter throughout the whole review process. Consequently, the findings from a rapid review are more likely to be applicable and relevant to the decision-making process. The rapid review also allows for balancing the interest in different types of effectiveness outcomes related to FIT programs and the limited timeframe to conduct the review and support timely decision-making. The Preferred Reporting Items for Systematic Reviews and Meta-Analyses (PRISMA) guided the review process and reporting of this work [25].

### Search strategy and study selection

With the support of content experts, a research librarian developed the search strategy based on PICO guidelines. Key search terms used were fecal immunochemical test, colorectal cancer, neoplasm, and screening. The search was completed on September 22, 2022, and conducted in three databases: Ovid Medical Literature Analysis and Retrieval System Online (MEDLARS Online/MEDLINE), Embase, and EBSCOhost Cumulated Index to Nursing and Allied Health Literature (CINAHL). Additional file 1 presents the search strategy used in Ovid MEDLINE. Full data search strategies for all databases are available upon request.

Inclusion criteria were as follows: quantitative or mixed-methods studies, systematic reviews and meta-analyses reporting on effectiveness of FIT programs in improving CRC screening among populations with no regular healthcare provider or described or identified by the papers’ authors as experiencing limited healthcare system access (e.g., rural residents, racialized communities, sexual and gender minorities). The main outcomes of interest were related to effectiveness of FIT programs: request or receipt of FIT kit; completion rates of FIT screening (returning of FIT kits with stool sample, FIT screening results available); and participation rates in follow-up colonoscopy after FIT positive results (referral or scheduling for colonoscopy and completion of colonoscopy). Another outcome of interest was acceptance of the FIT program. We included studies published from inception to September 22, 2022 and conducted in United Nations developed countries or in seven selected Organization for Economic Co-operation and Development (OECD) member countries. The full search strategy is available upon request from the corresponding author. Additional file 2 presents the inclusion and exclusion criteria, including the list of countries.

We used a systematic review software, Covidence [26], to perform title/abstract and full-text screening. Through a dual independent screening approach, two reviewers assessed titles and abstracts for a random selection of 10% of the studies retrieved in the search. A third reviewer helped resolve discrepancies as needed. This process was repeated until an inter-rater agreement of 100% was reached. At that point, the same two reviewers separately completed the primary screening of remaining studies. This same process was conducted for full-text screening. We performed reference-list screening of all included studies.

### Data extraction and analysis

One reviewer extracted data using a standardized data extraction tool. A second reviewer verified data to avoid incomplete information or misinterpretation. Using deductive and inductive approaches, a thematic analysis was conducted to guide evidence synthesis on effectiveness. The reviewers utilized the Cochrane Methods Equity PROGRESS-Plus [27] to classify findings by social factors affecting FIT program uptake. PROGRESS-Plus is an acronym used for identification of the following social factors: Place of Residence; Race/ethnicity/culture/language; Occupation; Gender/sex; Religion; Education; Socioeconomic Status; Social Capital; and Plus (which refers to any other factors not included in the previous components, such as age and disability).

### Quality appraisal

The Effective Public Healthcare Panacea Project (EPHPP) *Quality Assessment Tool for Quantitative Studies* [28] was used to assess the quality of methods used in the included studies. Two reviewers independently performed a quality appraisal of 10% of the included studies to assure consistency in scoring. Both reviewers met to resolve discrepancies through discussion and consensus; a third reviewer was present to resolve conflicts and support determination of the final score. The two reviewers then proceeded to complete the independent assessment of all remaining papers. None of the included papers were excluded due to a poor quality assessment score [29].

## Results

### Search outcomes

**Fig. 1:**
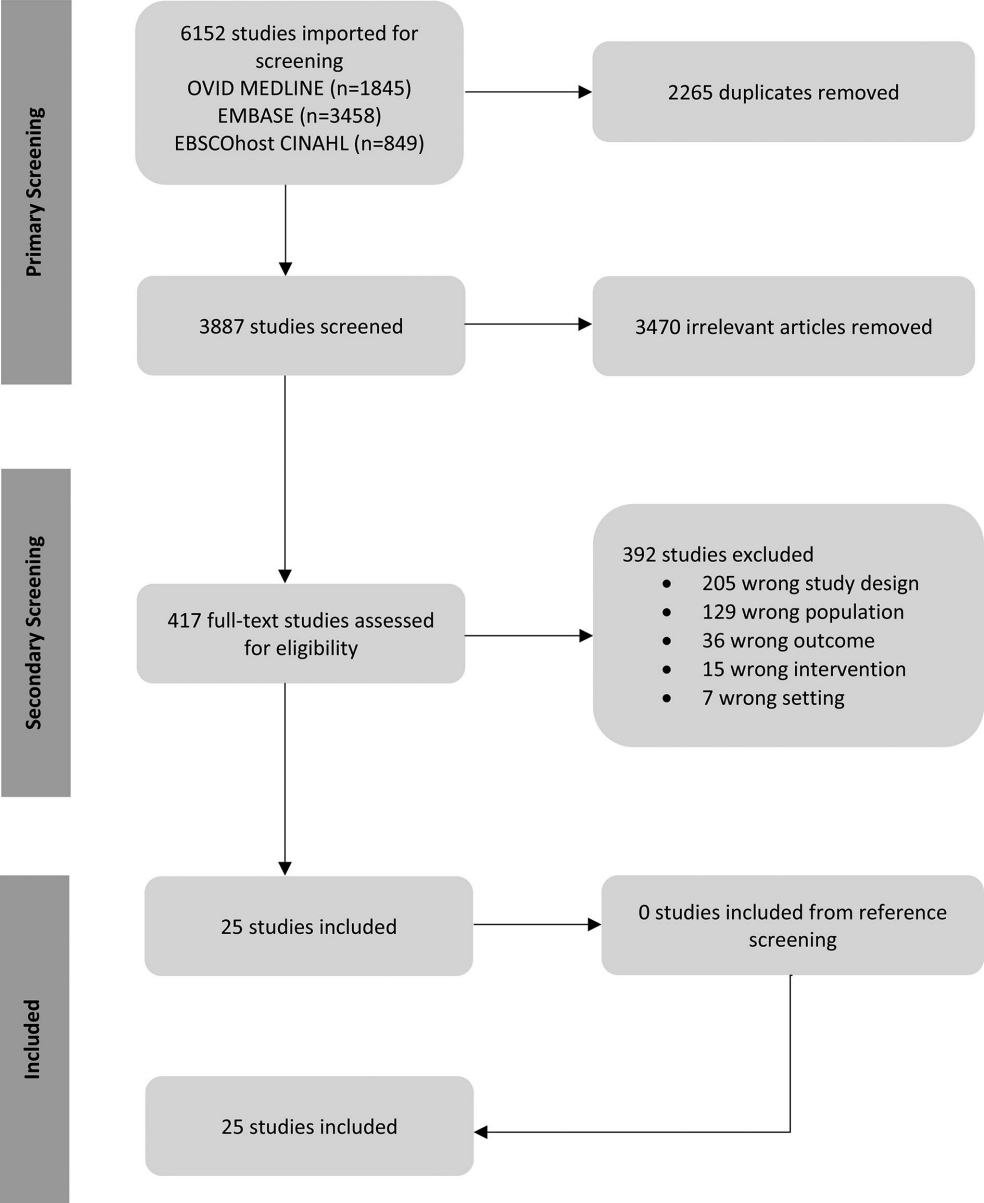
PRISMA chart of rapid review screening process

The search retrieved 6152 studies, from which 2265 duplicates were removed. Out of 3887 studies assessed during primary screening, 417 met the inclusion criteria and underwent full-text assessment. This rapid review resulted in 25 primary studies for data extraction [30–54]. No additional studies identified during the reference-list screening process met the inclusion criteria (Fig. 1).

Table 1 summarizes the main characteristics of included studies. Ten studies were published between 2021 and 2022; 14 studies investigated interventions in the United States; and the most common study designs used were randomized controlled trials and controlled clinical trials (n = 14). Most studies (n = 17) described the included populations as having no or limited access to the healthcare system (e.g., immigrant populations [52], uninsured groups [38–40], and publicly funded safety-net health system in United States [42, 43]). Only five studies stratified findings for the population group with no regular healthcare providers. Most studies examined the effectiveness of FIT kit delivered in-person (n = 10) or by mail (n = 13). Most studies on FIT effectiveness had FIT kits returned by mail (n = 12). In the quality appraisal, 16 studies scored as weak [31, 33, 37, 38, 40–42, 44–49, 51, 52, 54]; eight scored as ‘moderate’ [30, 32, 34, 36, 39, 43, 50, 53]; and one [35] as ‘strong’. Overall, the quality of evidence was weak due to parameters related to selection bias, study design, and reporting of follow-up rates. Additional file 3 provides a detailed summary of each of the included studies.

**Table 1:** Descriptive summary of the included studies in the rapid review

Characteristics	Categories	Number (n); Proportion (%)	References
Date of Publication	2011–2015	n = 3; 12%	Hillyer et al. 2011; Hillyer et al. 2014; Turrin et al. 2015
	2016–2020	n = 12; 48%	Bartholomew et al. 2019; Christy et al. 2016; Clarke et al. 2016; Crosby et al. 2017; Davis et al. 2016; Hirko et al. 2020; Lee et al. 2020; Sali et al. 2016; Sali et al. 2018; Somsouk et al. 2019; Stone et al. 2019; Symonds et al. 2019
	2021–2022	n = 10; 40%	Botteri et al. 2022; De Klerk et al. 2022; Gomes et al. 2021; Gupta et al. 2022; Lee et al. 2022; Lucas et al. 2021; Prakash et al. 2022; Ritzenthaler et al. 2022; Van der Meulen et al. 2022; Young et al. 2021
Location	United States	n = 14; 56%	Christy et al. 2016; Crosby et al. 2017; Davis et al. 2016; Gupta et al. 2022; Hillyer et al. 2011; Hillyer et al. 2014; Hirko et al. 2020; Lee et al. 2020; Lee et al. 2022; Lucas et al. 2021; Prakash et al. 2022; Ritzenthaler et al. 2022; Somsouk et al. 2020; Stone et al. 2019
	Italy	n = 3; 12%	Sali et al. 2016; Sali et al. 2018; Turrin et al. 2015
	Netherlands	n = 2; 8%	De Klerk et al. 2022; Van der Meulen et al. 2022
	Australia	n = 2; 8%	Symonds et al. 2019; Young et al. 2021
	Other	n = 4; 16%	Bartholomew et al. 2019; Botteri et al. 2022; Clarke et al. 2016; Gomes et al. 2021
Study Design^a^	Randomized controlled trials (with or without two arms) and controlled clinical trials	n = 14; 56%	Bartholomew et al. 2019; Botteri et al. 2022; Christy et al. 2016; Davis et al. 2017; Gupta et al. 2013; Hirko et al. 2020; Lee et al. 2020; Lee et al. 2022; Prakash et al. 2022; Sali et al. 2016; Sali et al. 2018; Somsouk et al. 2020; Symonds et al. 2019; Young et al. 2021
	Cohort (e.g., cohort: one group pre + post; cohort analytic: intervention and comparison group pre and post)	n = 4; 16%	Crosby et al. 2017; Hillyer et al. 2011; Ritzenthaler et al. 2022; Stone et al. 2019
	Ecological studies	n = 3; 12%	Clarke et al. 2016; de Klerk et al. 2022; Van der Meulen et al. 2022
	Cross-sectional studies	n = 2; 8%	Gomes et al. 2021; Turrin et al. 2015
	Other (e.g., retrospective record review)	n = 2; 8%	Hillyer et al. 2014; Lucas et al. 2021
Access to Healthcare Provider or Healthcare System	No access to regular healthcare provider (based on stratified data analysis)	n = 5; 20%	Crosby et al. 2017; Christy et al. 2016; Davis et al. 2017; Gomes et al. 2021; Stone et al. 2019
	No or precarious access to the healthcare system	n = 17; 68%	Bartholomew et al. 2019; Botteri et al. 2022; Gupta et al. 2013; Hirko et al. 2020; Hillyer et al. 2011; Hillyer et al. 2014; Lee et al. 2020; Lucas et al. 2022; Prakash et al. 2022; Ritzenthaler et al. 2022; Lee et al. 2022; Sali et al. 2016; Sali et al. 2018; Somsouk et al. 2022; Symonds et al. 2019; Turrin et al. 2015; Young et al. 2021
	Access to healthcare system not specified but results divided by SES^b^	n = 3; 12%	Clarke et al. 2016; de Klerk et al. 2022; Van der Meulen et al. 2022
Method of FIT Delivery	In-person	n = 10; 40%	Christy et al. 2016; Crosby et al. 2017; Davis et al. 2017; Gomes et al. 2021; Hillyer et al. 2011; Hillyer et al. 2014; Prakash et al. 2022; Sali et al., 2016; Sali et al. 2018; Stone et al. 2019
	By mail	n = 13; 52%	Bartholomew et al. 2019; Botteri et al. 2022; Clarke et al. 2016; Gupta et al. 2013; Hirko et al. 2020; Lee et al. 2022; Lee et al. 2020; Lucas et al. 2021; Ritzenthaler et al. 2022; Somsouk et al. 2022; Van der Meulen et al. 2022; Symonds et al., 2019; Young et al. 2021
	Unclear	n = 2; 8%	De Klerk et al. 2022; Turrin et al. 2015
Method of FIT Return	In-person	n = 3; 12%	Crosby et al. 2017; Sali et al.2016; Sali et al. 2018
	By mail	n = 12; 48%	Botteri et al. 2022; Christy et al. 2016; Davis et al. 2017; Gupta et al. 2013; Hirko et al. 2020; Lucas et al. 2022; Prakash et al. 2022; Ritzenthaler et al. 2022; Somsouk et al. 2022; Stone et al. 2019; Symonds et al. 2019; Young et al. 2021
	In-person or by mail	n = 3; 12%	Hillyer et al. 2011; Hillyer et al. 2014; Lee et al. 2022
	Unclear	n = 7; 28%	Bartholomew et al. 2019; Clarke et al. 2016; De Klerk et al. 2022; Gomes et al. 2021; Lee et al. 2020; Turrin et al. 2015; Van der Meulen et al. 2022

^a^ The study design is presented as articulated by the CHC team, which aligns with the parameters from the Effective Public Healthcare Panacea Project (EPHPP) *Quality Assessment Tool for Quantitative Studies* [28] as well as methodological knowledge

^b^ In these studies, participants with lower SES were included within the disadvantaged population group definitions

### Evidence synthesis

Through a thematic analysis, we identified three intervention design-related themes, which are interconnected. We present findings on effectiveness across these themes as follows.

### Delivery of culturally-tailored programs

Findings were mixed about effectiveness of culturally-tailored programs in increasing FIT uptake [30, 32, 35, 36, 39–42, 44, 46, 49]. Successful strategies supporting FIT participation among disadvantaged populations included engaging bilingual, lay health educators to support patient navigation [39, 40], employing language and interpretive services [42, 49], delivering FIT materials written in languages other than English [39, 40, 49], and using low-literacy and/or wordless instructions on how to complete testing [39, 40, 49]. Verbal, written, and visual instructions on how to collect and send the specimen to the laboratory were indicated as some of the reasons the intervention achieved a high rate of CRC screening (80.8%) [35]. However, studies [31, 35, 38, 47, 49, 52, 53] examining on participation in a colonoscopy after a positive FIT found interventions with verbal and written communication available in different languages were unsuccessful in ensuring participants with an abnormal FIT completed a colonoscopy. Two of those studies [35, 38] found 20–25% of participants with abnormal FIT results did not complete colonoscopies for reasons including comorbidities, health concerns, refusal, moving away, and failure to respond.

Interventions utilizing motivational messages on FIT screening improved participation, particularly in medically-underserved communities (24.6% among those who received the message at least once versus 3% among those who did not receive the message) [46] and rural areas (7.6% increase in intervention group vs. control group) [41]. Motivational interviewing techniques (discussing the relevance of CRC screening and exploring participants’ feelings about the test) were embedded in live telephone outreach with trained program champions [46] and motivational messaging (highlighting the preventability of CRC and ease and affordability of FIT screening) was incorporated into a mailed invitation letter that addressed barriers in screening [41]. In a study on loss-framed messaging (i.e., emphasizing the life costs of not being screened), participants receiving culturally-targeted loss-framed messaging (addressing cultural needs) were significantly more receptive to obtaining CRC screening compared to standard loss-framing [44].

Conversely, a study found that tailored educational resources had a negative impact: 12.3% and 8.3% absolute reduction in screening participation when sending a targeted promotion digital video disc (DVD; featuring Māori elders and a famous rugby player) to the Māori and the Pacific ethnicity groups, respectively [30]. Similarly, the FIT completion rates were 78.1% in a group receiving a targeted, low-literacy, photonovella booklet and DVD and 83.5% in a group receiving only a CRC screening brochure [35]. Another study reported the FIT kit return was only 81.9% in the group receiving a culturally-targeted CRC photonovella booklet plus a FIT kit; that proportion was 90.3% in the group receiving a standard CRC screening brochure plus a FIT kit [32].

Only two tailored studies [30, 49] described the viability of FIT returns. Partially due to the guidance provided on how to perform the test, promotional DVDs on FIT process resulted in the return of fewer spoiled tests (for Māori, 12.4% versus 33.1% in the no DVD group; for Pacific group were 21.9% versus 42.1% in the no DVD group) [30]. Damaged or lost FIT kit were indicated as a reason for 61.8% of participants not returning the FIT sample after reminder phone calls to address their concerns [49].

### Method of delivery for FIT: mail-out and in-person

Most included studies delivered FIT kits by mail [30, 31, 33, 38, 41–44, 46, 49, 51, 53, 54], with others offering in-person [32, 34, 35, 37, 39, 40, 45, 47, 48, 50]. Findings were mixed regarding the effectiveness of each approach; this was largely context- and population-dependent. Studies [38, 46, 49] reported that mailing FIT kits to participants resulted in higher return rates (ranging, across studies, from 22.4% [46] to 57.9% [49]) than when FIT kits were provided at regular healthcare visits (ranging, across studies, from 12.1% [38] to 37.4%% [49]).

Among people who previously completed FIT, mailed FIT outreach resulted in higher subsequent FIT completion rates when compared to the usual care group, which included coaching, reminder calls by providers (83.9% versus 71.8%) [42]. Among patients with no history of FIT, the completion rates - although were relatively modest - indicated the mailed FIT outreach intervention was also successful in that population (52.5% in the outreach group versus 37.2% in the usual care group) [42].

Studies that randomized participants into a group being mailed a FIT kit and a group being invited to a CRC blood test found no significant differences in uptake (37.8% versus 36.9%, *p* > 0.05 [51]; 12.0% versus 13.3%, *p* = 0.884 [54]). When a choice was provided, participants opted to receive a FIT kit by mail over completing a blood test (9.7% versus 3.8%, *p* = 0.005) [54]. A mailed outreach invitation to complete FIT at no-cost (40.7%) was more effective than mailed outreach invitations to a no-cost colonoscopy (24.6%) [38].

Along with FIT mail-out intervention, additional strategies were used to improve the FIT completion rate. Uptake increased when using a mailed motivational invitation letter for screening, followed by mailing a cost-free FIT kit in comparison to the group receiving standard invitation letter with no kit (30.1% versus 22.5%) [41]. FIT completion rates were higher among participants who received at least one live interaction via phone call compared to those who had none (27.8% versus 10.5%, *p* < 0.001) [46]. However, voicemails [46], educational materials and DVDs [30], and advanced notification phone calls [43] were unsuccessful in increasing the return of mailed-out FIT kits. Across the studies on mail-out FIT programs [30, 31, 33, 38, 41–44, 46, 49, 51, 53, 54], the most common method used to return the FIT kits was through a postage paid return envelope [31, 38, 41, 44, 46, 51, 54].

A longitudinal analysis found organized mailed FIT outreach involving a mailed postcard and a phone call prior to mailing a FIT, and then up to two phone call reminders had a high cumulative FIT completion over a period of 2.5 years among participants with a history of prior FIT completion (83.9% versus 71.8% in the usual care group) [42]. Other study that included only population with a history of FIT completion found modest differences in FIT completion between the group receiving notification phone calls and the group with no phone calls [43]. This suggests that groups with prior history of FIT may require less reminder methods for the subsequent FIT completion [43]. Studies examining return rates of mailed-out FIT for participants who had previously completed a FIT [42, 43, 49] found the intervention group was more likely to complete a subsequent FIT (e.g., 71.9% versus 35.7% in the group with no prior test, *p* < 0.001) [49].

Studies with in-person FIT kit delivery intervention [32, 34, 35, 37, 39, 40, 45, 47, 48, 50] showed high rates of FIT completion rate (ranging, across studies, from 71.3 to 90.0%) [32, 34, 35, 39, 45, 50]. In-person interaction between participants and research or healthcare staff who provided CRC education and demonstration of FIT specimen collection procedure using a free FIT was indicated as a reason for the success [34, 35, 37, 39, 40].

A study that adopted home visits for in-person delivery of FIT kits concluded that approach was effective, achieving a 71.3% FIT sample return rate via mail among low-income people and medically-underserved communities [50]. In-person delivery of FIT reduced the need for multiple follow-ups, with 87% kits returned within two weeks after the delivery [39]. Those with in-person return [34] were as successful as studies with a postage paid envelope to return the FIT [32, 45] or studies that provided both options to participants [39, 40].

In a study, referral rates to optical colonoscopy (OC) were compared between participants in an in-person FIT delivery program and participants who underwent a computed tomography colonography (CTC). It found higher referral rates to OC among participants with positive results in CTC than among those who tested positive to FIT (10% versus 5.5%) [47]. A study combining CRC screening with other cancer screening procedures reported that delivering a FIT kit in-person with a mammography was feasible and efficacious in improving return of FIT kits (90%) [39].

### Follow-up reminders

Methods to remind participants to complete FIT sample collection and return FIT kits were generally successful in increasing CRC screening rates [46, 49, 50]; for instance, completion rates were 24.6% in the group with at least one successful contact via telephone compared to 3.0% for no contact [46]. Live reminders [46, 49, 50] (e.g., follow-up or reminder phone calls) helped address participants’ concerns and improved rates of request or receipt of FIT kits, but not necessarily return rates [49]. In comparison to combined, multiple reminder methods, spaced out telephone reminders (e.g., every two weeks or one month apart) contributed to improved return rates (e.g., 16.8% increase with reminders every two weeks for 60 days, *p* < 0.01) [45] and prevented over-communication for participants who do not want multiple reminders [45].

### Equity considerations

Given multiple social factors influence access to healthcare system, we applied equity considerations lenses to understand what barriers this disadvantaged population group faces and what strategies may respond better to their needs. Below are the equity considerations organized by the social components of the PROGRESS-Plus framework [27]. There were no findings for occupation.

### Place of residence

Closer proximity (a digital map estimate of driving time from patients’ home address to the screening centre) [31, 32] where the FIT kit was being sent from/to had a positive impact on FIT participation rates (e.g., 61.2% among those living within 20-minute-driving distance vs. 56.1% among those living within 40-minute-driving distance to the screening centre, *p* < 0.01) [31].

Lack of access to healthcare services and information in rural areas may contribute to low FIT uptake [37]. A motivational letter accompanying the FIT kit [41] and engagement or personal contact with a service provider for the FIT kit delivery [34] may increase participation among rural populations. One study found people living in areas with high levels of urban density were the least likely to participate in FIT programs [36]. Those authors suggest that, given that large cities have more medical facilities, residents can more often access medical care compared to those in the smaller cities or rural areas. Therefore, they recommend such jurisdictions to adopt FIT screening to better respond to the barriers the residents face to healthcare access [36].

### Race/ethnicity/culture/language

Several studies reported FIT participation among different races/ethnicities [31, 35, 38, 40, 45, 49, 50, 52]. Regular reminders and postcards supported participation of medically-underserved minorities [45, 49]. Delivering FIT programs in a non-medical setting may have successfully reduced cultural differences and cultural barriers centered around the medical community [50]. Compared to native populations, participation rates in FIT screening (51.3% versus 34.3%, *p* < 0.001) [52] and colonoscopy follow-up after positive FIT (94.3% versus 88.7%, *p* < 0.01) [31] among immigrant populations was typically lower. Compared to the native population, the low compliance among immigrants might be due to their high mobility resulting in mail invitations being sent to outdated addresses [31, 52].

Language tailoring (e.g., multiple languages, visuals, culturally framed) and resources offered through trusted sources (e.g., community members, lay health advisors) supported FIT participation [32, 39–42, 44, 49]. For instance, studies [39, 49] found that availability of interpreter services in languages other than English resulted in high screening rates among disadvantaged populations (90.0% [39] and 57.9% [49]) because of the linguistic and cultural identification between staff and participants. Visual brochures (e.g., photonovella approaches) [32] or motivational letters [41] incorporating cultural preferences, language, and appropriate literacy levels may have increased uptake of FIT.

### Gender and/or sex

The impact of gender and/or sex on FIT participation was not consistent across the studies. In some studies [31, 33, 40, 41, 47, 48, 52], females were more likely than males to participate in FIT (rates ranging, across studies, from 22.9% [48], to 85.4% [41]). Other studies found no difference in return rates of FIT kits between males and females [37, 45, 50]. This contrasts with findings from van der Meulen et al. [53] that showed sex as a significant variable in FIT participation across the socioeconomic gradient. For individuals who had been previously screened for CRC, participation rates were similar for both males and females [52].

Receipt of a DVD on FIT kit had a larger negative impact on return rates among males than females. For example, for the male Māori population, the difference in FIT kit return between no DVD and DVD groups was 16.4%, whereas, for the Māori females, that difference was only 9.4% [30]. Factors influencing males’ participation in FIT programs included fear of being diagnosed with cancer, fatalism, lack of knowledge, and being misinformed. For females, factors preventing participation included negative attitudes, beliefs and emotions, and the impact of social influences [33].

### Religion

The association between religious beliefs and not returning FIT kits (Adjusted OR: 1.09, 95% CI: 1.02–1.16, *p* = 0.015) [32] were attributed to beliefs that God(s) is(are) responsible for one’s health outcomes [32].

### Education

An overall recommendation was to develop materials and resources tailored for participants with lower literacy [32, 33, 35, 39, 49]. In unadjusted (crude) analyses in some studies, individuals with higher levels of education had lower FIT participation rates [34, 37]. However, after adjustment for other factors (e.g., age, sex) there was no significant difference in participation. However, when education was controlled for other factors (e.g., age and sex), there was no significant difference in participation [34, 37]. One study showed that level of education was not associated with FIT kit return [50].

### Socioeconomic status

While two studies reported no significant difference in FIT participation by SES [37, 51], other studies [36, 47, 53, 54] found that participants from higher socioeconomic status (SES) groups were more likely to complete a FIT test (e.g., 76.2% least disadvantaged group versus 23.8% most disadvantaged group) [54]. Low participation rates in the mail-out FIT programs were attributed to low SES [31, 33, 53]. The lower screening uptake in deprived areas could be partially explained by the inverse association between deprivation and health literacy (where lower health literacy is expected in areas experiencing higher deprivation) [33]. In one study where the entire population was from a low socioeconomic area, it was found that even within this group there was a higher participation rate among the disadvantaged group (54%, RR: 1.06, 95% CI: 1.04–1.08, *p* < 0.001) when compared with the most disadvantaged group (46%) [33]. Technology-related barriers including not having a phone number (4.1% versus 23.9% with a phone number, *p* < 0.01) or voicemail (11.8% versus 26.2% with a voicemail, *p* < 0.001) resulted in significantly lower FIT participation among socioeconomically disadvantaged groups [46].

One study [48] indicated higher participation rates among higher SES participants (25.7% high SES versus 18.0% low SES, *p* = 0.009) that completed FIT after non-participation in a reduced-preparation CTC. However, there was no difference in participation by SES for participants who completed FIT after full-preparation CTC and after optical colonoscopy screening. To mitigate lower participation rates within lower SES groups, it was suggested that FIT programming be delivered among disadvantaged population groups living in urban clusters (e.g., housing complexes, housing for families) to support FIT uptake [50]. Lastly, compliance in follow-up colonoscopy after a positive FIT was low among those in the lowest SES (75.8% versus 81.3% highest SES; OR = 0.73, 95% CI 0.69–0.77) [53].

### Social capital

A participant’s connection to community and social networks were predictors of FIT completion. African-Americans living in large public housing developments (e.g., senior homes) were 1.87 times (95% CI: 0.987–3.552) more likely to return FITs than residents living in other types of housing (e.g., private rental units, non-complex housing) [50].

### Plus

Age was a factor impacting FIT participation. While one study found no association, [50] another study reported an increasing trend in FIT participation rates with age [54]. Two studies [47, 48] showed higher participation for individuals over the age of 60 years (e.g., 54% in 61–65 age group versus 48% in 54–60 age group, *p* < 0.001) [47], with another study specifically indicating that the 65–69 age group was more likely to participate in FIT programs than age group 50–54 years (52.8%, adjusted prevalence ratio = 1.45, 95% CI: 1.20–1.76, *p* < 0.01) [37].

One study found that, for the oldest group, FIT participation was higher among the native-born participants than immigrant participants (49.2% versus 25.8%); in contrast, for the youngest group, higher participation rates were recorded among the immigrant group (74.2% versus 50.8% in the native-born group) [52]. This difference can be potentially explained by younger immigrant groups being better “assimilated” and being easier to reach when compared with the older aged immigrant group [52].

One study identified disability as imposing a barrier to FIT completion. FIT kit return rates were lower for individuals experiencing a disability/unable to work (73%) compared with individualswho were not employed outside the home (87%) (e.g., unemployed, students, and homemakers) [32]. Perception of health and weight issues also influenced FIT participation. Those who did not self-reported as being overweight or obese were 1.95 times (95% CI: 1,07-3.55, *p* = 0.029) more likely to return FIT [34]. Similarly, the FIT return was lower among participants with obesity (57.3%) than those with normal BMI (67.4%) [41]. Those reporting fair or poor perceived health (79.3%) showed higher rates of participation than those reporting better self-rated health (65.6%) [50].

## Discussion

This review synthesized evidence on the effectiveness of FIT programs in increasing CRC screening among disadvantaged groups with no regular healthcare provider or limited healthcare system access. We summarized findings across three intervention design-related themes: Delivery of Culturally-tailored Programs; Method of Delivery for FIT: Mail-out and In-person; and Follow-up Reminders. Findings could inform (re)design and implementation of large-scale interventions to improve FIT uptake among this target population.

Overall, culturally-tailored programs involving communication strategies (e.g., specific messages crafted with plain language and translated into different languages; participation of lay health educators; motivational messages) may increase the effectiveness of FIT programs. This is consistent with other research describing language and literacy as structural barriers compromising patient navigation, and thus access to health services [23].

While it remains unclear if mail-out or in-person FIT delivery was more effective, we found that the use of additional strategies along with each mode of delivery may increase FIT kit return rates. For example, a motivational screening letter, a cost-free FIT kit, and live phone interaction should be implemented in mail-out programs. For in-person delivery, demonstration of how to collect FIT sample and home visits may better meet the needs of the populations with limited healthcare access. Despite only one study combining FIT program with other cancer screening programs, its success in CRC screening echoes recommendations elsewhere of integration of preventative cancer procedures for opportunistic screening [55]. However, opportunistic screening should not replace organized FIT screening programs to ensure universal invitation and equitable participation of all eligible patients.

As part of a strategy to increase community demand for CRC screening, reminders have successfully alerted patients and increased screening rates [56, 57]. Reminders were mainly effective in increasing rates of request and receipt of FIT kits, but not necessarily return rates. Our findings showed that reminders may be less effective in increasing the level of FIT among population groups with prior FIT completion screening. This may signal a good retention rate in the FIT program. It is also important to consider that live reminders may be cost-prohibitive as they require intensive interactions with patients. While automatic notification may be an alternative, it may fail to address patients’ fears and concerns [57].

Uptake of follow-up colonoscopy among participants with abnormal FIT results varied across studies (from 14.8% [47] to 93.3% [31])– this variability was reported elsewhere [57]. As improving FIT screening rates may not ensure care continuity, adequate infrastructure [57] becomes critical to ensure patients are aware of the benefits of undergoing subsequent colonoscopy and have easy access to follow-up care.

This population group with no regular healthcare provider or limited access to the healthcare system experiences multiple, intersecting disadvantages that perpetuate and increase barriers to healthcare system access. Recognizing that, we used a specific tool [27] to distill the social determinants of health affecting FIT uptake. Our review uncovered social factors that may reduce people’s participation of FIT screening programs, which aligns with the literature [58, 59]. Findings suggest decision-makers and healthcare practitioners should consider the needs and priorities of specific social groups (e.g., religious groups) when designing intervention strategies. Intentional targeting and tailoring of the interventions to the populations’ identities and local contexts are needed for equitable participation in universal FIT programs.

Our review has some limitations. Our population criterion specified the inclusion of studies targeting populations without regular healthcare providers or describing their populations as medically-underserved or experiencing disadvantages regarding healthcare access. We had to rely on the information provided by those studies, which was often vague or unclear. As such, the study team met regularly to discuss the inclusions. However, there is still uncertainty about the population’s lack of or limited access to regular healthcare providers or to the healthcare system. In some studies, authors identified populations as medically-underserved; however, recruitment occurred in healthcare settings (e.g., health clinics) [33, 38, 40, 42, 43, 45, 46, 49] or employed community-based strategies [30, 31, 36, 40, 44, 47, 48, 51–54]. Similarly, in other studies it was unclear whether or not *having an assigned family doctor* meant having a regular healthcare provider (see, for instance, Gomes et al. 2021 [37]). A limitation in the included studies was the different meanings for the term ‘uptake’ as defined by study authors. For example, uptake could refer to the collection/receiving FIT kit or the kit return or completion of FIT (see, for instance, Clarke et al. 2016 [33] and Bartholomew 2019 [30], respectively). To avoid misinterpretation, we recorded terms as presented by the original authors. Due to the heterogeneity of the study design and definition of the main outcomes, we were unable to perform a meta-analysis. The included studies had important methodologic limitations that preclude conclusions concerning effectiveness (only eight papers were scored as of moderate quality, and one, as strong). Lastly, given that most of the findings came from studies using randomized control trial and controlled clinical trial designs, we acknowledge evaluating implementation and effectiveness outcomes in real world settings were out-of-scope. Their findings do not discuss the policy and administrative-practice implications for planning and implementation of real-world public health interventions, such as local population’s needs, costs and resource requirements, scalability and sustainability of the programs, and organizational factors associated with the health system context.

The strengths of this rapid review include: a comprehensive search strategy to account for the nuance of the language around healthcare system access; quality appraisal; use of rigorous and systematic methods for screening and assessment; and a detailed analysis on social determinants of health affecting the effectiveness of FIT programs.

Overall, our findings contribute to the literature in which most reviews on FIT programs have thus far focused on either general population [19, 21, 57, 60] or specific socially disadvantaged groups, like rural populations and low-income populations [20, 61]. To the best of our knowledge, this is the first review on the topic investigating this specific population group. Another unique aspect of this review is the multidimensional analysis of the FIT programs. We examined closely the multiplicity of factors– from features of the programs to the social background and identities of the patients– and their interconnections that may influence the success in achieving the health goals set by the CRC screening initiatives. Enriched with the use of PROGRESS-Plus framework [27], our review gathered the evidence that may be an indicative of what has worked, for whom, and under what circumstances. This is a critical knowledge in informing (re)design and implementation of population-wide, equity-informed programs in real-world settings.

## Conclusion

Our review presented evidence-based strategies that may be more successful in improving FIT screening rates among population group who do not have access to regular healthcare providers or have limited access to healthcare system. Multicomponent interventions combined with tailored strategies may be more effective among this population who may be at high risk of CRC due to their limited opportunities to access preventive healthcare services.

The population group who does not have regular healthcare provider or has limited access to the healthcare system is very diverse and has systematically experienced cumulative disadvantages for their identities and backgrounds. Understanding their unique priorities and needs and recognizing the interplay of social determinants and factors across the healthcare system are critical steps in the efforts to improve access to FIT programs and other universal health initiatives. Evaluation studies on the implementation of CRC screening programs in real-world settings are needed to provide evidence on best strategies for spreading and scaling-up intervention approaches. The use of implementation science models and frameworks will enhance the implementation and evaluation approaches.

### Electronic supplementary material

Below is the link to the electronic supplementary material.

**Additional File 1**: MEDLINE Search Strategy

**Additional File 2**: Inclusion and exclusion criteria

**Additional File 3**: Data summary of included studies in the rapid review

## Data Availability

The datasets used and/or analysed during the current study are available from the corresponding author on reasonable request.

## Abbreviations

CI: Confidence Interval
CRC: Colorectal Cancer
CINAHL: Cumulated Index to Nursing and Allied Health Literature
DVD: Digital Video Disc
EPHPP: Effective Public Healthcare Panacea Project
FOBT: Fecal Occult Blood Test
FIT: Fecal Immunochemical Test
gFOBT: Fecal Occult Blood Test
GP: General Practitioner
IARC: International Agency for Research on Cancer
MEDLARS Online/MEDLINE: Medical Literature Analysis and Retrieval System Online PICO: Patient/Population, Intervention, Comparison, and Outcomes
PRISMA: Preferred Reporting Items for Systematic Reviews and Meta-Analyses
PROGRESS-Plus: Place of Residence, Race/Ethnicity/Culture/Language, Occupation, Gender/sex, Religion, Education, Socioeconomic status, Social capital, and Plus (e.g., Personal characteristics associated with discrimination, Features of relationships, and Time-dependent relationships)
OECD: Organization for Economic Co-operation and Development
SES: Socioeconomic Status

## References

[1] International Agency for Research on Cancer (IARC). Colorectal cancer screening. 2019. 300 p. 31985915

[2] Ferlay J, Ervik M, Lam F, Colombet M, Mery L, Piñeros M et al. Global Cancer Observatory: Cancer Today. [Internet]. Lyon, France: Internaional Agency for Research on Cancer 2020 [Available from: https://gco.iarc.fr/today/about.

[3] Fitzpatrick-Lewis D, Ali M, Warren R, Kenny M, Sherifali D, Raina P (2016). Screening for Colorectal Cancer: a systematic review and Meta-analysis. Clin Colorectal Cancer.

[4] Health Quality Council of Alberta. Patient completion of screening tests [Internet]. 2019. Available from: https://focus.hqca.ca/primaryhealthcare/screening/.

[5] Heitman SJ, Hilsden RJ, Au F, Dowden S, Manns BJ (2010). Colorectal cancer screening for average-risk North americans: an economic evaluation. PLoS Med.

[6] Coldman A, Flanagan W, Nadeau C, Wolfson M, Fitzgerald N, Memon S (2017). Projected effect of fecal immunochemical test threshold for colorectal cancer screening on outcomes and costs for Canada using the OncoSim microsimulation model. J Cancer Policy.

[7] Charters TJ, Strumpf EC, Sewitch MJ (2013). Effectiveness of an organized colorectal cancer screening program on increasing adherence in asymptomatic average-risk canadians. BMC Health Serv Res.

[8] Adhikari K, Yang H, Teare GF (2022). Patterns of up-to-date status for colorectal cancer screening in Alberta: a cross-sectional study using survey data. Can Med Association Open Access J.

[9] Davis MM, Renfro S, Pham R, Lich KH, Shannon J, Coronado GD (2017). Geographic and population-level disparities in colorectal cancer testing: a multilevel analysis of Medicaid and commercial claims data. Prev Med.

[10] Zapka JG, Puleo E, Vickers-Lahti M, Luckmann R (2002). Healthcare system factors and colorectal cancer screening. Am J Prev Med.

[11] Hughes AE, Tiro JA, Balasubramanian BA, Skinner CS, Pruitt SL, Social Disadvantage (2018). Healthcare utilization, and Colorectal Cancer Screening: leveraging longitudinal patient address and Health Records Data. Cancer Epidemiol Biomarkers Prev.

[12] He E, Lew J-B, Egger S, Banks E, Ward RL, Beral V (2018). Factors associated with participation in colorectal cancer screening in Australia: results from the 45 and up study cohort. Prev Med.

[13] Venturelli F, Sampaolo L, Carrozzi G, Working Group PASSI, Zappa M, Rossi PG (2019). Associations between cervical, breast and colorectal cancer screening uptake, chronic diseases and health-related behaviours: data from the Italian PASSI nationwide surveillance. Prev Med.

[14] Clarke RB, Therkildsen C, Gram MA, Andersen KK, Mørch LS, Tybjerg AJ (2020). Use of primary health care and participation in colorectal cancer screening–a Danish national register-based study. Acta Oncol.

[15] Sibley L, Weiner J (2011). An evaluation of access to health care services along the rural-urban continuum in Canada. BMC Health Serv Res.

[16] Canadian Institute for Health Information. Population grouping methodology [information sheet] [Internet]. Ottawa, ON: CIHI.; 2017. Available from: https://www.cihi.ca/sites/default/files/document/Infosheet-PopGroupMethod-2020-en.pdf.

[17] McDonald JT, Conde H (2010). Does geography matter? The health service use and unmet health care needs of older canadians. Can J Aging/La Revue Canadienne Du Vieillissement.

[18] Wong ST, Regan S (2009). Patient perspectives on primary health care in rural communities: effects of geography on access, continuity and efficiency. Rural Remote Health.

[19] Mohan G, Chattopadhyay SK, Ekwueme DU, Sabatino SA, Okasako-Schmucker DL, Peng Y (2019). Economics of Multicomponent interventions to increase breast, cervical, and Colorectal Cancer Screening: A Community Guide systematic review. Am J Preventative Med.

[20] Davis MM, Freeman M, Shannon J, Coronado GD, Stange KC, Guise J-M (2018). A systematic review of clinic and community intervention to increase fecal testing for colorectal cancer in rural and low-income populations in the United States– How, what and when?. BMC Cancer.

[21] Dougherty MK, Brenner AT, Crockett SD, Gupta S, Wheeler SB, Coker-Schwimmer M (2018). Evaluation of interventions intended to increase Colorectal Cancer Screening Rates in the United States: a systematic review and Meta-analysis. JAMA Intern Med.

[22] Sabatino SA, Lawrence B, Elder R, Mercer SL, Wilson KM, DeVinney B (2012). Effectiveness of interventions to increase screening for breast, cervical, and colorectal cancers: nine updated systematic reviews for the guide to community preventive services. Am J Prev Med.

[23] Community Preventive Task Force. Increasing Colorectal Cancer Screening: Multicomponent Interventions [Internet]. Community Preventive Task Force; 2016 Available from: https://www.thecommunityguide.org/media/pdf/Cancer-Screening-Multicomponent-Colorectal.pdf.

[24] The Community Guide. Increasing Colorectal Cancer Screening: Multicomponent Interventions [Internet]. n.d. Available from: https://www.thecommunityguide.org/findings/cancer-screening-multicomponent-interventions-colorectal-cancer.

[25] Page MJ, McKenzie JE, Bossuyt PM, Boutron I, Hoffmann TC, Mulrow CD (2021). The PRISMA 2020 statement: an updated guideline for reporting systematic reviews. BMJ.

[26] Veritas Health Innovation. Covidence systematic review software. Melbourne, Australia. n.d.

[27] Cochrane Methods Equity. PROGRESS-Plus Cochrane Methods Equityn.d. Available from: https://methods.cochrane.org/equity/projects/evidence-equity/progress-plus.

[28] Effective Public Healthcare Panacea Project. Quality Assessment Tool for Quantitative Studies n.d. Available from: https://www.ephpp.ca/quality-assessment-tool-for-quantitative-studies/.

[29] Garritty C, Gartlehner G, Kamel C, King V, Nussbaumer-Streit B, Stevens A (2021). Cochrane Rapid Reviews Interim Guidance from the Cochrane. J Clin Epidemiol.

[30] Bartholomew K, Zhou L, Crengle S, Buswell E, Buckley A, Sandiford P (2019). A targeted promotional DVD fails to improve Māori and Pacific participation rates in the New Zealand bowel screening pilot: results from a pseudo-randomised controlled trial. BMC Public Health.

[31] Botteri E, Hoff G, Randel KR, Holme Ø, de Lange T, Bernklev T (2022). Characteristics of nonparticipants in a randomised colorectal cancer screening trial comparing sigmoidoscopy and faecal immunochemical testing. Int J Cancer.

[32] Christy SM, Davis SN, Williams KR, Zhao X, Govindaraju SK, Quinn GP (2016). A community-based trial of Educational interventions with Fecal Immunochemical Test for Colorectal Cancer Screening Uptake among blacks in Community settings. Cancer.

[33] Clarke N, McNamara D, Kearney PM, O’Morain CA, Shearer N, Sharp L (2016). The role of area-level deprivation and gender in participation in population-based faecal immunochemical test (FIT) colorectal cancer screening. Preventative Med.

[34] Crosby RA, Stradtman L, Collins T, Vanderpool R (2017). Community-based Colorectal Cancer Screening in a Rural Population: who returns fecal immunochemical test (FIT) kits?. J Rural Health.

[35] Davis SN, Christy SM, Chavarria EA, Abdulla R, Sutton SK, Schmidt AR (2017). A randomized controlled trial of a multicomponent, targeted, low-literacy educational intervention compared with a nontargeted intervention to boost colorectal cancer screening with fecal immunochemical testing in community clinics. Cancer.

[36] de Klerk CM, van der Vlugt M, Smagge BA, Toes-Zoutendijk E, Lansdorp-Vogelaar I, Dekker E (2022). Urban density differences in colorectal cancer screening participation and screening yield in the Netherlands. Preventative Med Rep.

[37] Gomes FS, Kislaya I, Seabra D, Cordeiro E, Nunes B (2021). Factors Associated with the Use of Fecal Immunochemical tests and Colonoscopy in the INSEF Portuguese Population. Portuguese J Public Health.

[38] Gupta S, Halm EA, Rockey DC, Hammons M, Koch M, Carter E (2013). Comparative effectiveness of Fecal Immunochemical Test Outreach, Colonoscopy Outreach, and Usual Care for boosting Colorectal Cancer Screening among the Underserved: a Randomized Clinical Trial. JAMA Intern Med.

[39] Hillyer GC, Basch CE, Schmitt KM, Neugut AI (2011). Feasibility and efficacy of pairing fecal immunochemical testing with mammography for increasing colorectal cancer screening among uninsured Latinas in northern Manhattan. Preventative Med.

[40] Hillyer GC, Schmitt KM, Freedberg DE, Kramer RA, Su Y, Rosenberg RM (2014). Fecal-based Colorectal Cancer Screening among the Uninsured in Northern Manhattan. Am J Preventative Med.

[41] Hirko KA, Lennon SA, Lucas T, Miller DC, Jimbo M, Leibfritz SJ (2020). Improving Colorectal Cancer Screening in a rural setting: a randomized study. J Preventative Med.

[42] Lee B, Keyes E, Rachocki C, Grimes B, Chen E, Vittinghoff E (2022). Increased Colorectal Cancer Screening sustained with mailed fecal immunochemical test Outreach. Clin Gastroenterol Hepatol.

[43] Lee B, Patel S, Rachocki C, Issaka R, Vittinghoff E, Shapiro JA (2020). Advanced notification calls prior to mailed fecal immunochemical test in previously screened patients: a Randomized Controlled Trial. J Gen Intern Med.

[44] Lucas T, Thompson HS, Blessman J, Dawadi A, Drolet CE, Hirko KA (2021). Effects of culturally targeted message framing on Colorectal Cancer Screening among African americans. Health Psychol.

[45] Prakash S, Merza N, Hosseini O, Ward H, Mansi T, Balducci M, et al. Increasing Fecal Immunochemical Test Return Rates by implementing effective reminder to complete kit communication with participants: a Quality Improvement Study. Cureus. 2022;14(5). 10.7759/cureus.25169. 10.7759/cureus.25169 PMC9206862 35746986

[46] Ritzenthaler D, Deshpande S, Ryan M, Daprano J (2022). Colorectal Cancer screening with mailed fecal immunochemical tests and Telephone Outreach at a Community Health Center during the COVID-19 pandemic. J Health Care Poor Underserved.

[47] Sali L, Mascalchi M, Falchini M, Ventura L, Carozzi F, Castiglione G (2016). Reduced and full-Preparation CT Colonography, Fecal Immunochemical Test, and Colonoscopy for Population Screening of Colorectal Cancer: a Randomized Trial. J Natl Cancer Inst.

[48] Sali L, Ventura L, Mascalchi M, Falchini M, Mantellini P, Delsanto S (2018). Faecal immunochemical test in subjects not attending screening computed tomography colonography and colonoscopy in a randomized trial. Eur J Cancer Prev.

[49] Somsouk M, Rachocki C, Mannalithara A, Garcia D, Laleau V, Grimes B (2020). Effectiveness and cost of Organized Outreach for Colorectal Cancer Screening: a Randomized, Controlled Trial. J Natl Cancer Inst.

[50] Stone R, Stone JD, Collins T, Barletta-Sherwin E, Martin O, Crosby R (2019). Colorectal Cancer Screening in African American HOPE VI Public Housing residents. Fam Community Health.

[51] Symonds EL, Hughes D, Flight I, Woodman R, Chen G, Ratcliffe J (2019). A Randomized Controlled Trial Testing Provision of Fecal and Blood Test options on participation for Colorectal Cancer Screening. Cancer Prev Res.

[52] Turrin A, Zorzi M, Rossi PG, Senore C, Campari C, Fedato C (2015). Colorectal cancer screening of immigrants to Italy. Figures from the 2013 National Survey. Prev Med.

[53] van der Meulen MP, Toes-Zoutendijk E, Spaander MC, Dekker E, Bonfrer JM, van Vuuren AJ (2022). Socioeconomic differences in participation and diagnostic yield within the Dutch national colorectal cancer screening programme with faecal immunochemical testing. PLoS ONE.

[54] Young GP, Chen G, Wilson CJ, McGrane E, Hughes-Barton DL, Flight IH (2021). Rescue of nonparticipants in Colorectal Cancer Screening: a randomized controlled trial of three noninvasive Test options. Cancer Prev Res.

[55] Weiss JM, Pandhi N, Kraft S, Potvien A, Carayon P, Smith MA (2018). Primary care colorectal cancer screening correlates with breast cancer screening: implications for colorectal cancer screening improvement interventions. Clin Translational Gastroenterol.

[56] Lam WW, Murphy CC, Vernon SW,‘Colorectal Cancer Screening’, in, Breitbart W and others, editors. Psycho-Oncology, 4 edn. 2021; online edn, Oxford Academic. 2021.

[57] Issaka RB, Avila P, Whitaker E, Bent S, Somsouk M (2019). Population health interventions to improve colorectal cancer screening by fecal immunochemical tests: a systematic review. Prev Med.

[58] Dressler J, Johnsen AT, Madsen LJ, Rasmussen M, Jorgensen LN (2021). Factors affecting patient adherence to publicly funded colorectal cancer screening programmes: a systematic review. Public Health.

[59] Clarke N, Sharp L, Osborne A, Kearney PM (2015). Comparison of uptake of colorectal cancer screening based on fecal immunochemical testing (FIT) in males and females: a systematic review and meta-analysis. Cancer Epidemiol Biomarkers Prev.

[60] Jager M, Demb J, Asghar A, Selby K, Mello EM, Heskett KM (2019). Mailed Outreach is Superior to Usual Care alone for Colorectal Cancer Screening in the USA: a systematic review and Meta-analysis. Dig Dis Sci.

[61] Wang H, Roy S, Kim J, Farazi PA, Siahpush M, Su D (2019). Barriers of colorectal cancer screening in rural USA: a systematic review. Rural Remote Health.

