# Supplementary material for: Effective strategies for Fecal Immunochemical Tests (FIT) programs to improve colorectal cancer screening uptake among populations with limited access to the healthcare system: a rapid review
Source: BMC Health Serv Res. 2024 Jan 23;24:128. doi: 10.1186/s12913-024-10573-4 (PMC10807065; doi:10.1186/s12913-024-10573-4)
Supplement: Supplementary file 1 — Additional File 1: MEDLINE Search Strategy [file 12913_2024_10573_MOESM1_ESM.docx]

## **Additional File 1. MEDLINE Search Strategy**

MEDLINE search strategy (adapted for other databases)

1. (fecal immuno* adj2 test*).mp
2. (faecal immuno* adj2 test*).mp.
3. (Diagnostic Self Evaluation/ or "Direct-to-Consumer Screening and Testing"/ or "Early Detection of Cancer"/ or Mass Screening/ or Self-Test/) and (Adenomatous Polyposis Coli/ or Anal Gland Neoplasms/ or Anus Neoplasms/ or Colitis-Associated Neoplasms/ or Colonic Neoplasms/ or Colorectal Neoplasms, Hereditary Nonpolyposis/ or Colorectal Neoplasms/ or Gardner Syndrome/ or Rectal Neoplasms/ or Sigmoid Neoplasms/) and (self* or home? or mail* or kit?).mp.
4. (self* or screen* or rescreen* or "early detect*" or home? or mail* or kit? or FIT) adj4 (((colorectal or colon* or colitis* or anus or anal or Sigmoid or bowel* or rectal or rectum or gastrointestin* or intestine* or ileum or jejunum) adj2 (cancer* or precancer* or neoplasm* or carcinoma* or dysplas* or dyskaryos* or tumor* or tumour* or malignanc* or adenocarcinoma* or lesion* or squamous or small cell or large cell)) or gardner syndrome* or adenomatous polyposis coli)).ab.
5. ((self* or screen* or rescreen* or "early detect*" or home? or mail* or kit? or FIT) adj4 (((colorectal or colon* or colitis* or anus or anal or Sigmoid or bowel* or rectal or rectum or gastrointestin* or intestine* or ileum or jejunum)) adj2 (cancer* or precancer* or neoplasm* or carcinoma* or dysplas* or dyskaryos* or tumor* or tumour* or malignanc* or adenocarcinoma* or lesion* or squamous or small cell or large cell)) or gardner syndrome* or adenomatous polyposis coli).ti,kf,kw.
6. or/1-5 [FIT TESTS]
7. limit 6 to English language
8. limit 7 to (comment or editorial or letter or news or newspaper article or preprint or webcast)
9. 7 not 8
