# Supplementary material for: Effective strategies for Fecal Immunochemical Tests (FIT) programs to improve colorectal cancer screening uptake among populations with limited access to the healthcare system: a rapid review
Source: BMC Health Serv Res. 2024 Jan 23;24:128. doi: 10.1186/s12913-024-10573-4 (PMC10807065; doi:10.1186/s12913-024-10573-4)
Supplement: Supplementary file 2 — Additional File 2: Inclusion and exclusion criteria [file 12913_2024_10573_MOESM2_ESM.docx]

**Additional File 2. Inclusion and exclusion criteria.**

| **Characteristic** | **Inclusion Criteria** | **Exclusion Criteria** |
| --- | --- | --- |
| **Language** | Full text is published in English. |  |
| **Date** | None. |  |
| **Peer-Reviewed** | Study is published in a peer-reviewed journal. |  |
| **Study Type** | Primary research studies using quantitative or mixed methods, e.g., experimental studies, quasi-experimental studies, and observational studies (cohort, cross-sectional, and case-control studies) reporting effectiveness.  Descriptive quantitative studies reporting effectiveness.  Systematic reviews and meta-analyses containing primary studies within the inclusion criteria. | Primary research studies using qualitative methods and analysis.  Cost-effectiveness studies.  Any other types of review that are not systematic reviews or meta-analyses, such as scoping reviews and narrative reviews.  Studies in the form of comments, editorials, letters to editor, theoretical papers, books, book chapters, protocols, case studies, case reports, grey literature (e.g., magazine articles, dissertations, doctoral theses, conference papers, position statements, pre-prints).  Systematic reviews and meta-analyses including other types of study designs (outlined in exclusion criteria above) will be excluded, even if findings are reported separately for included study designs. |
| **Location of Intervention** | United Nations (UN) developed economies and 7 selected Organization for Economic Co-operation and Development (OECD) member countries.  UN developed economies: Australia, Austria, Belgium, Bulgaria, Canada, Croatia, Cyprus, Czech Republic, Denmark, Estonia, Finland, France, Germany, Greece, Hungary, Iceland, Ireland, Italy, Japan, Latvia, Lithuania, Luxembourg, Malta, Netherlands, New Zealand, Norway, Poland, Portugal, Romania, Slovakia, Slovenia, Spain, Sweden, Switzerland, United Kingdom, and United States.  7 OECD member countries: Chile, Colombia, Costa Rica, Israel, South Korea/Republic of Korea, Mexico and Turkey. | None. |
| **Settings** | No limitation on settings. This includes, but is not limited to, healthcare settings and community-based settings. |  |
| **Population** | Population may be identified by authors as not having a regular healthcare provider (e.g., family doctor, personal physician, nurse) or may be described or identified as disadvantaged, underserved, and/or vulnerable in terms of access to the healthcare system. *Note:* Disadvantaged, underserved, and/or vulnerable groups can be identified by authors or inferred based on the likelihood that certain populations may not have a regular healthcare provider (e.g., Indigenous groups; people living in rural, remote or underserved communities; hard-to-reach populations; immigrants; refugees; visible minorities; 2SLGBTQ+; low SES individuals).  Age: any eligible age group for CRC-FIT screening of the included jurisdiction.  Asymptomatic populations (i.e., not exhibiting symptoms of CRC or not have been diagnosed with CRC). | Populations with a regular healthcare provider.  The target population or participants are not described or identified as disadvantaged, underserved, and/or vulnerable in relation to access to healthcare system.  The target population is described as a general and/or entire population (i.e., not recruiting or sampling for a specific group) with no further subgrouping or stratification in analysis for specific disadvantaged, underserved, and/or vulnerable populations.  Populations who are symptomatic and/or received a diagnosis of CRC. |
| **Intervention** | Fecal Immunochemical Testing (FIT).  Interventions may be delivered by healthcare organization(s) and government (national, provincial/territorial, regional or local level) or in partnerships with organizations. | Interventions that are not part of the FIT program or any other types of CRC screening (e.g., colonoscopy, flexible sigmoidoscopy, blood biomarkers, DNA tests, traditional 3-sample fecal occult blood test/Guaiac-based test (gFOBT), fecal deoxyribonucleic acid, etc.) |
| **Comparisons** | Any, where relevant.  Effectiveness can be compared:  - to the national/local/or population average that has been predetermined.  - to other screening tools (FOBT, DNA, etc.) |  |
| **Outcomes** | Effectiveness outcomes should be related to the FIT program uptake for colorectal cancer screening. It may include:  - program uptake (initiation/taking FIT tests, acceptance of the program)  - completion rates of screening (uptake and return FIT tests)  - FIT participation rates or scheduling of colonoscopy, referral to colonoscopy, and completion of colonoscopy after FIT results. | Outcomes that do not report on effectiveness.  Outcomes that are related to receiving or referrals to the treatment of CRC. |
| **Other** | Full publication is available. |  |
