# Supplementary material for: Effective strategies for Fecal Immunochemical Tests (FIT) programs to improve colorectal cancer screening uptake among populations with limited access to the healthcare system: a rapid review
Source: BMC Health Serv Res. 2024 Jan 23;24:128. doi: 10.1186/s12913-024-10573-4 (PMC10807065; doi:10.1186/s12913-024-10573-4)
Supplement: Supplementary file 3 — Additional File 3: Data summary of included studies in the rapid review [file 12913_2024_10573_MOESM3_ESM.docx]

## **Additional File 3. Data summary of included studies in the rapid review.**

| **First Author’s Name, Year Published**  **Location**  **Objective of Study** | **Study Design^a^**  **Recruitment**  **Intervention**  **Tailoring Strategies** | **Population Size**  **Population Demographics** | **Outcomes of Interest (Program Uptake; Completion Referral, Acceptance, Barriers, and Facilitators)** | **Author Reported Limitations**  **Quality Appraisal** |
| --- | --- | --- | --- | --- |
| Bartholomew et al., 2019  New Zealand  Examined the impact of mailed invitation FIT screening program in Māori and Pacific ethnicity populations. | Pseudo-randomized trial design (controlled clinical trial)  All residents between the ages of 55-74 of the Waitematā area were recruited to the Bowel Screening Program. Only Māori, Pacific, and Asian ethnicity invitees were given follow-up calls and reminder letters.  Participants were randomized to either the intervention group (DVD group) that received a promotional DVD on FIT with a reminder to complete a FIT or a control group (no DVD group) that only received reminder letters to complete a FIT.  Tailoring   - Incorporated important Māori community figures (e.g., a famous rugby player and local Māori elders) to educate and promote colorectal cancer screening. | *Population size:* 5,271 participants (n=2,333 Māori; n=2,938 Pacific)  *Age (Māori):* 50-54 39.0%; 55-59 27.5%; 60-64 16.8%; 65-69 10.7%; 70+ 5.4%; missing 0.7%  *Age (Pacific):* 50-54 33.9%; 55-59 24.9%; 60-64 19.1%; 65-69 12.4%; 70+ 8.7%; missing 1.0%  *Sex (Māori):* Female 59.0%; Male 40.2%; Missing 0.7%  *Sex (Pacific):* Female 54.6%; Male 44.4%; Missing 1.0%  *Deprivation Quintile (Māori):* Q1 (least) 12.2%; Q2 14.9%; Q3 19.5%; Q4 25.8%; Q5 (most) 17.2%; missing 10.4%  *Deprivation Quintile (Pacific):* Q1 (least) 5.3%; Q2 8.7%; Q3 17.5%; Q4 32.8%; Q5 (most) 25.7%; missing 10.0% | FIT Uptake   - For Māori group,13.6% participated in the DVD group, and 25.9% participated in the no DVD group. - For Pacific ethnicity groups,10.1%% participated in the DVD group, and 18.4% participated in the no DVD group. - There was significantly more participation in the Māori group than the Pacific ethnicity groups in the return of FIT when being sent a DVD (p=0.043). - Overall, being sent a DVD had a larger negative effect on participation (Māori CI: 12.3%, 9.1%-15.5%; Pacific ethnicity: 8.3%, 5.8%-10.8%) - With the DVD group, there were fewer spoiled FITs (Māori DVD 12.4%; Māori no DVD 33.1%; Pacific ethnicity DVD 21.9%; Pacific ethnicity no DVD 42.1%).   Barriers   - Reduced participation in the DVD group could be due to the content being uncomfortable or problematic for participants. - The education DVD protagonist was a woman, which may have discouraged some men. - Other potential barriers were no ownership of a DVD player, lack of time to play the DVD, and the feeling of having to watch the DVD before performing the test. | No randomization at an individual level; and lack of information if participants watched the DVD provided in the DVD group.  Moderate |
| Botteri et al. 2022  Norway  Identified the demographic, socioeconomic, and comorbidity factors associated with non-participation to FIT and sigmoidoscopy screening. | Randomized trial (randomized trial with no control group)  Participants between ages of 50-74 were identified through a population registry belonging to two geographical regions of Norway. The randomly selected people were invited to participate in either FIT or sigmoidoscopy.  Individuals were mailed-out an invitation letter based on the intervention arm they were randomized to: FIT or sigmoidoscopy. The FIT arm also received a sampling kit. FITs were mailed-out and a reminder message was sent to participants with no response. | *Population Size:* 116,938 invited participants (n=46,919 sigmoidoscopy; n=70,019 FIT); n=40,931 participated in FIT  *Age:* 50-55 17.5%; 56-60 22.4%; 61-65 21.7%; 66-70 22.5%; 70+ 16.0%  *Sex:* Female 53.2%; Male 46.8%  *Immigration Background:* Norwegian 93.1%; Immigrant 6.9%  *Marital Status:* Cohabit/married 79.4%; Single/widowed 20.3%  *Education:* Primary school 17.3%; High school 46.8%; 1-4 years university 25.3%; >4 years university 10.1%  *Occupation:* Employed 62.9%; Retired 36.6%; Unemployed 0.5%  *Income*: ≤484,000 19.0%; 484,001-755,000 25.4%; 755,001-1,130,000 27.1%; >1,130,000 28.6%  *Note:* Population demographics reported for participants in the FIT intervention only. | FIT Uptake   - 58.5% of individuals participated in the first round of FIT. - After two rounds of FIT, participation in at least one of the two rounds was 64.7%. - Low FIT uptake was associated with younger age, male gender, low education, unemployment or retirement, low income, living alone, immigrant status, living further away from screening center, and use of antidiabetic and psychotropic drugs.   Referral   - Low colonoscopy follow-up after a positive FIT was associated with immigrant status, living further from the screening centre, and individuals on antidiabetic and psychotropic drugs.   Barriers   - Low SES and immigrants may perceive greater barriers in accessing screening centres for sigmoidoscopy and, thus, may prefer FIT, which can be completed at home. | Lack of information on previous colonoscopies; and no information on health behaviours (e.g., smoking, body mass index, alcohol intake, or dietary habits).  Weak |
| Christy et al., 2016  United States  Examined the efficacy of a culturally targeted colorectal screening intervention, including brochure+FIT and photonovella+FIT to increase screening uptake among Black individuals. | Screening trial (controlled clinical trial)  Participants were recruited through newspapers, online ads, face-to-face interactions, and referrals through peers and previously enrolled participants of another study.  Four Tampa Bay regions were randomly assigned to either the photonovella+FIT or brochure+FIT groups. The participants in the photonovella+FIT group received a photonovella booklet, in which a story was told using photos and limited texts. The participants in the brochure+FIT group received standard brochures from Centers for Disease Control (CDC) and Prevention “Screen for Life” brochures.  Tailoring   - Lay advisors and primary care providers collaborated to create the photonovella. - Photonovella was used to match the cultural and literacy levels of participants. - To appeal to immigrants, the use of multiple United States flags and Afro-Caribbean flags were used. - FITs were provided at no charge. | *Population Size:* 330 participants (n=144 photonovella+FIT; n=186 Brochure+FIT)  *Sex (photonovella+FIT)*: Female 41.0%; Male 59.0%  *Sex (Brochure+FIT)*: Female 52.7%; Male 47.3%  *Ethnicity (photonovella+FIT:* Hispanic 1.4%; Non-Hispanic 98.6%  *Ethnicity (Brochure+FIT:* Hispanic 3.8%; Non-Hispanic 96.2%  *Racial Heritage (photonovella+FIT):* African-American 97.2%; Caribbean/Haitian/other 2.8%  *Racial Heritage (Brochure+FIT):* African-American 90.3%; Caribbean/Haitian/other 9.7%  *Marital Status (photonovella+FIT):* Married/partnered 32.6%; Divorced/separated/widowed 36.1%; Never married/single 31.2%  *Marital Status (Brochure+FIT):* Married/partnered 29.6%; Divorced/separated/widowed 35.5%; Never married/single 34.9%  *Education (photonovella+FIT)*: Less than high school/GED 22.9%; High school/GED 32.6%; Some college 27.8%; College graduate/post-graduate 16.7%  *Education (Brochure+FIT)*: Less than high school/GED 11.8%; High school/GED 34.9%; Some college 36.0%; College graduate/post-graduate 17.2%  *Insurance (photonovella+FIT)*: Yes 43.8%; No 56.3%  *Insurance (Brochure+FIT)*: Yes 57.0%; No 43.0%  *Occupation (photonovella+FIT)*: Employed 34.7%; Not employed 22.2%; Retired 13.9%; Unable to work 29.2%  *Occupation (Brochure+FIT)*: Employed 44.1%; Not employed 25.8%; Retired 16.7%; Unable to work 13.4%  *Has a regular healthcare provider (photonovella+FIT)*: Yes 43.8%; No 55.6%  *Has a regular healthcare provider (Brochure+FIT)*: Yes 62.4%; No 37.6%  *Income (photonovella+FIT)*: Less than $10,000 43.1%; $10,000-$25,000 27.8%; $25,001-$35,000 6.9%; $35,001-$50,000 9.7%; $50,001-$75,000 4.9%; $75,001-$100,000 0.7%; $100,001+ 2.1%  *Income (Brochure+FIT)*: Less than $10,000 32.3%; $10,000-$25,000 31.7%; $25,001-$35,000 10.8%; $35,001-$50,000 11.8%; $50,001-$75,000 7.0%; $75,001-$100,000 1.6%; $100,001+ 1.1%  *Prior CRC screening test (photonovella+FIT)*: Yes 22.2%; No 77.8%  *Prior CRC screening test (Brochure+FIT)*: Yes 33.3%; No 65.6%  *Have an annual physical exam (photonovella+FIT):* Yes 50.7%; No 49.3%  *Have an annual physical exam (Brochure+FIT):* Yes 59.7%; No 38.7% | FIT Uptake   - Within six months, 81.9% of FITs were returned in the photonovella+FIT group and 90.3% in the brochure+FIT group. There was no significant difference in FIT returns between the two groups. - No significant difference between screening uptake or time of FIT return between the photonovella+FIT group and brochure+FIT group. - 40% of participants did not have a regular provider, of which there were no significant differences in uptake of FIT between the photonovella+FIT and brochure+FIT groups.   Facilitators   - Providing cost-free FIT may have been more important than the use of culturally-targeted education materials. | Lack of randomization at an individual level; unequal group sizes and baseline differences; small number of participants reporting foreign-born Black racial heritage status even though the study was targeting a specific racial group; selection bias in participant recruitment; and limited generalizability to Black people living in a single geographic region in the United States.  Moderate |
| Clarke et al., 2016  Ireland  Examined the effect of sex and deprivation on FIT screening uptake. | Not specified. (Ecological study)  Residents of Tallaght between ages of 50-74 were identified through seven primary care practices and invited to participate in FIT.  The Tallaght Hospital and Trinity College Dublin Cancer Screening Programme offered two rounds of biennial screening which included a FIT, an intervention letter, information on colorectal cancer (CRC) screening, and the Irish Cancer Society help-line number. Screening programs were free for all participants and if cancer was detected, treatment was covered. | *Population size:* 9,785 invited participants (n=5,451 participated; 55.7%)  *Age*: <60 34.7%; 60-64 29.7%; 65-69 23.1%; 70-74 11.9%; 75+ 0.8%  *Sex*: Female 56.6%; Male 43.4%  *Deprivation*: Very disadvantaged 10.1%; Disadvantaged 30.1%; Marginally below average 46.6%; Marginally above average 10.8%; Affluent 2.4% | FIT Uptake   - Overall FIT participation was 60%. - Average age of FIT participation was 62 years old. - FIT uptake was higher in participants over the age of 60 compared to those aged less than 60. - FIT uptake was significantly lower in males than females (p<0.001). - The strongest socio-demographic predictor of FIT uptake in population-based screening was deprivation. - There was a significant difference in uptake in disadvantaged areas compared to very disadvantaged areas. FIT may be a better screening tool in more deprived areas (p<0.001). - Overall, FIT uptake was significantly lower in males compared to females (p <0.001). | Inability to modify variables, lack of information on CRC screening history; and inability to determine the effect of multiple invitations to households.  Weak |
| Crosby et al., 2017  United States  Investigated factors associated with the return rate of FIT in rural populations. | Not specified (cohort one group)  Participants were recruited in rural Kentucky counties through flyers posted in the local health department. Additional community outreach (senior citizen centers, health and wellness events) and direct referrals from local health clinics.  Participants had direct contact with research staff who were trained to describe the FIT to participants.  Tailoring   - Personal contact in FIT distribution was implemented. | *Population size:* 345 participants  *Age:* average 57.2 years old (SD=11.07), ranging from 30 to 75 years old  *Sex:* Female 64.8%; Male 35.2%  *Ethnicity:* mostly White; 3 identified as Black; 2 identified as Native American  *Marital status:* Married 45.8%  *Education:* Graduated high school 69.6%  *Insurance:* Yes 89.6%; No 9.0%; Not available 1.4%  *Insurance types:* Medicaid 32.3%; Medicare beneficiaries 38.4%; Not available 29.3%  *Has a regular healthcare provider: Yes* 79.4%; No 18.8%; Not available 1.8%  *Overweight or obesity:* Yes 50.4%; No 48.1%; Not available 1.5%  *Personal income less than $15,000 annually:* Yes 51.3%; No 48.7%  *Smokers:* Yes 34.5%; No 63.8%; Not available 1.7% | FIT Uptake   - 82% of participants returned the FIT. - Bivariate analysis showed that participants without regular healthcare providers were more likely to return their FIT (92.3%) than participants with a regular provider (79.9%). However, this relationship was not retained in the multivariate analysis. - Participants with an annual income of less than US$15,000 (OR=2.85; 95% CI: 1.56-5.24; P < .001) and those who self-report as not being overweight or obese (OR=1.95; 95% CI: 1.07-3.55; P =.029) were more likely to return their FIT. | Convenience sampling of the rural population; no generalization to other rural populations; and unknown confounders not selected within studies that may impact data analysis.  Moderate |
| Davis et al., 2017  United States  A Randomized Controlled Trial of a Multicomponent, Targeted, Low-Literacy, Educational Intervention Compared with a Nontargeted Intervention to Boost Colorectal Cancer Screening with Fecal Immunochemical Testing in Community Clinics | Randomized controlled trial (controlled clinical trial)  Racial and ethnically diverse researchers approached potential participants in clinic waiting areas to recruit participants.  Participants were randomly assigned to either receive the Colorectal Cancer Awareness, Research, Education, and Screening trial (CARES) or a standard intervention. In the CARES intervention, participants received a photonovella booklet and DVD accompanied by an in-person FIT collection demonstration. In the standard intervention, participants received a Centers for Disease Control (CDC) brochure accompanied by an in-person FIT collection demonstration. Both written and verbal instructions and in-person FIT collection demonstrations were available for all participants.  Tailoring   - Education intervention (photonovella booklet and DVD) was developed to address limited health literacy. It utilized local characters, content, storylines, and photos. | *Population size:* 417 participants (n=207 standard intervention; n=210 CARES intervention)  *Sex (Standard):* Female 53.4%; Male 46.6%  *Sex (CARES):* Female 53.8%; Male 46.2%  *Ethnicity (Standard):* Not Hispanic/Latino 89.3%; Hispanic/Latino 10.7%  *Ethnicity (CARES):* Not Hispanic/Latino 90.0%; Hispanic/Latino 10.0%  *Race (Standard)*: White 61.8%; Black 30.4%; Other 6.8%  *Race (CARES)*: White 70.5%; Black 25.7%; Other 4.8%  *Country of birth (Standard):* United States 94.2%; Outside of United States 5.8%  *Country of birth (CARES):* United States 90.9%; Outside of United States 9.1%  *Marital status (Standard):* Married/living with partner 32.5%; Separated/divorced/widowed 40.3%; Single/never married 27.2%  *Marital status (CARES):* Married/living with partner 29.5%; Separated/divorced/widowed 49.1%; Single/never married 21.4%  *Education (Standard):* Less than high school diploma 25.2%; High school diploma 38.3%; More than high school diploma 36.4%  *Education (CARES):* Less than high school diploma 22.8%; High school diploma 41.4%; More than high school diploma 35.7%  *Insurance (Standard)*: Yes 62.6%; No 37.4%  *Insurance (CARES)*: Yes 60.5%; No 39.5%  *Employment status (Standard):* Employed 20.0%; Not employed 52.0%; Retired/disabled 28.1%  *Employment status (CARES):* Employed 28.6%; Not employed 50.0%; Retired/disabled 21.4%  *Regular physician (Standard):* Yes 63.9%; No 36.1%  *Regular physician (CARES):* Yes 65.7%; No 34.3%  *Household income (Standard):* <$10,000 67.0%; >$10,000 33.0%  *Household income (CARES):* <$10,000 65.2%; >$10,000 34.8%  *Health literacy (Standard):* ≤6 (low) 35.9%; ≥7 64.1%  *Health literacy (CARES):* ≤6 (low) 36.2%; ≥7 63.8%  *Family history of cancer (Standard)*: Yes 48.1%; No 51.9%  *Family history of cancer (CARES)*: Yes 49.5%; No 50.5%  *Annual physical examination (Standard):* Yes 45.1%; No 54.9%  *Annual physical examination (CARES):* Yes 46.9%; No 53.1%  *Prior screening (any test; Standard):* Yes 32.2%; No 67.8%  *Prior screening (any test; CARES):* Yes 30.6%; No 69.4% | FIT Uptake   - Overall, FIT uptake for the study was 80.8% with no significant difference between the CARES and standard intervention (78.1% and 83.5% respectively). - There was no significant difference in time to FIT uptake between the CARES and standard intervention. - Compared to the standard intervention, uptake in the CARES was significantly lower in the racial/ethnic minority groups (p<0.005), those with a family history of cancer (p<0.05), those receiving annual physical examinations (p<0.05), and those with high cancer awareness (p<0.05). - Univariate analysis found that ownership of a health insurance (OR=2.14, 95% CI: 1.24-3.72, p<0.05), no access to a regular provider (OR=1.67; 95% CI: 1.02-2.75, p<0.05), younger ages (OR=1.07, 95% CI: 1.00-1.14, p≤0.05), and recruitment via a federally quality health clinic versus a primary care community clinic (OR, 1.67; 95% CI, 1.02-2.75, p<0.05) were significantly associated with no FIT returns. - Multivariate analysis showed that only participants with health insurance were less likely to return the FIT (adjusted OR= 2.10; 95% CI: 1.04-4.26, p<0.05).   Referrals   - Of the 21 of participants who had abnormal FIT results, only 16 completed colonoscopies. Five participants refused to complete colonoscopies.   Facilitators   - A facilitator to the higher uptake of FIT was the assurance that participants with no health insurance would be provided with a cost-free colonoscopy after an abnormal FIT. - Human interaction with study coordinators may have fostered trust and improved FIT uptake. | Combined interventions without stratified analysis taking into account the written and verbal instruction, collection demonstration, and types of education delivery; no comparison to a usual-care intervention; the possibility of patients not screened in this trial having sought CRC screening elsewhere; selection bias; focused on initial FIT uptake; and lack of generalizability as patient were recruited from a single geographic area.  Strong |
| de Klerk et al., 2022  Netherlands  Examined the participation rates of FIT program among lower SES individuals in urban dense locations. | Not specified (ecological study)  National CRC screening program that has age-specific roll-out. In 2014 and 2015, those aged 61, 63, 65, 67, 69, 75, and 76 were mailed a FIT.  National CRC screening program that mailed-out colorectal cancer screening participation letters (FIT) to participants’ homes every two years. | *Population size*: 1,873,639 invited participants (n=1,365,768 participants)  *Urban Density Level*: UD1 14.8%; UD2 24.8%; UD3 19.8%; UD4 21.2%; UD5 19.4%^^[[1]](#footnote-1)^^  *Participation by five levels of urban density and SES*: UD1 - SES1 65.5%, SES2 64.4%, SES3 63.8%, SES4 64.1%, SES5 58.6%  UD2 - SES1 73.4%, SES2 73.0%, SES3 72.6%, SES4 71.1%, SES5 57.6%  UD3 - SES1 75.8%, SES2 75.4%, SES3 75.0%, SES 4 74.9%, SES 5 70.0%  UD4 - SES1 76.7%, SES2 77.2% SES 3, 77.7% SES4 76.9% SES5 73.3%  UD5 - SES 1 76.6%, SES2 77.7%, SES3 77.1%, SES4 76.5%, SES5 73.8%^^[[2]](#footnote-2)^^ | FIT Uptake   - Participants in rural areas (UD5) had higher participation in FIT (77.3%) than high urban areas (UD1) (62.8%) (RR=1.23, 95% CI: 1.23-1.24). - Within all regions (UD5-UD1), participation was the lowest among those with the lowest SES. - Within those with the lowest SES, participation was lowest for those in very high urban densities. | Potential changes in participation over time because of increased awareness of the screening program or targeting younger age groups; exclusion of participants due to missing postal code; ecological fallacy.  Moderate |
| Gomes et al., 2021  Portugal  Examined factors associated with CRC screening (FIT and colonoscopy) in Portuguese populations aged 50-74 years. | Cross-sectional study (cross-sectional study)  Self-reported data came from the 2015 National Health Examination Survey (INSEF), which used a representative, probabilistic sample.  FIT is the primary screening test and is offered to all asymptomatic individuals aged 50-74 years. | *Population size:* 2,489 participants  *Age:* 50-54 24.3%; 55-59 20.0%; 60-64 22.1%; 65-69 18.7%; 70-74 15.0%  *Sex:* Female 53.5%; Male 46.5%  *Degree of urbanization:* rural 27.5%; urban 72.5%  *Education level:* elementary school 50.0%; middle school 28.3%; secondary school 12.0%; higher education 9.7%  *Employment status:* employed 37.7%; unemployed 10.0%; other 52.2%  *Economic capacity:* no 38.1%; yes 61.9%  *Assigned family doctor:* no 11.5%; yes 88.5% | FIT Uptake   - The age group 65-69 years presented the highest proportion of use of FIT compared to the age group 50-54 years (52.8%; adjusted prevalence ratio=1.45; 95% CI 1.20–1.76). - The use of FIT increased proportionally with age. - Higher use of FIT was found among those who have a family doctor (47.6%; aPR = 1.50; 95% CI 1.14-1.98). | Inability to establish causal relationship given the study design; no investigation of other external factors that may influence the uptake and use of FIT; reliance on self-reported data leading to potential biased information; and limited generalization to foreign populations given different cultural and social backgrounds, healthcare systems, and stage of implementation of national CRC screening programs.  Weak |
| Gupta et al., 2013  United States  Compared the effectiveness of a FIT mailed outreach program with a colonoscopy outreach program and usual care in an underserved population. | Randomized controlled trial (controlled clinical trial)  Participants were recruited from the John Peter Smith Health Network (JPS) that offers medical assistance for the uninsured population.  Participants were randomized and assigned to either the FIT outreach, colonoscopy outreach, or the usual care group. In both the outreach groups, participants were mailed-out an invitation to participate in a cost-free colorectal cancer screening and followed up with an automated phone message and two live reminder phone calls. The usual care group received clinic-based opportunistic screening (gFOBT, colonoscopy, barium enema, or sigmoidoscopy).  Tailoring   - Mailed letters contained both English and Spanish invitations with risk of colorectal cancer based on age. - Colorectal cancer screening was offered to participants as cost-free. - It provided aid for scheduling colonoscopy. | *Population size*: 5,970 participants (n=479 colonoscopy outreach; n=1,593 FIT outreach; n=3,898 usual care)  *Age*: Mean 59 years (SD=3)  *Sex:* Female 62.3%; Male 37.7%  *Race/ethnicity:* White 41.0%; Black 23.2%; Hispanic 27.9%; Other 7.8%  *Primary language:* English 83.0%; Spanish 17.0%  *Note:* Population demographics reported for participants in the FIT intervention only. | FIT Uptake   - 40.7% (95% CI: 38.3%-43.1%) of participants completed screening for FIT outreach, 24.6% (95% CI: 20.8%-28.5%) completed colonoscopy outreach, and 12.1% (95% CI: 11.1%-13.1%%) completed the usual care group. - ​​There were significant differences in participation for outreach programs versus usual care (p<0.001) within sex and race/ethnicity groups. - In comparison to the colonoscopy outreach, the FIT outreach program had significantly more uptake among men (p<0.001), women (p<0.001), whites (p<0.001), Blacks (p=0.001), and Hispanics (p<0.001).   Referral   - 60 patients were found to have abnormal FIT results, of which 49 completed subsequent colonoscopy. - The reasons for not completing colonoscopy included: competing health concerns, comorbidities, failure to respond, refusal of colonoscopy, and moving out of state. | Lack of generalizability due to strict inclusion criteria; screening participation based on only the first round of invitation; inability to study differences in CRC mortality; no investigation of feasibility of implementing this intervention; and absence of an intervention arm that would offer participants the possibility to choose between FIT or colonoscopy, which could lead to higher screening rates.  Weak |
| Hillyer et al. 2011  United States  Assessed the feasibility and efficacy of a program aimed at uninsured Latinas receiving mammography to improve colorectal cancer (CRC) screening. | Not specified. (cohort one group)  Uninsured Hispanic women were recruited in the Northern Manhattan Cancer Screening Partnership through community outreach.  All participants received one-on-one education sessions with lay health educators (10-12 minutes) that included a CRC education component and CRC screening education component. Participants also received written instructions in Spanish and English that included visuals on FIT specimen collection in addition to a kit that contained the test card, specimen brushes, disposal bags, labels, and pre-filled test request form. If the FIT was not returned within a 2-week timeframe, the participant received a telephone reminder (reminders were repeated every week for 8 weeks).  Tailoring   - All written instructions were presented in Spanish and English and at a 6th grade literacy level based on the Flesch-Kincaid method. - Lay health educators delivered the education sessions. | *Population size:* 197 women  *Age:* Mean 54.8 years (SD=3.8)  *Race:* White, Hispanic 82.2%; Black, Hispanic 16.2%; Hispanic, unknown race 1.0%; Hispanic, other race 0.5%  *Country of origin:* Dominican Republic 61.4%; Other 38.6%  *Marital status:* Married 30.5%; Living as married 4.8%; Widowed 5.9%; Separated 16.0%; Divorced 20.3%; Single, never married, or never lived as married 22.5%  *Education:* Less than 8^th^ grade 17.1%; 8^th^ to 11^th^ grade 20.3%; High school graduate or equivalent 24.1%; Vocational or trade school 7.5%; Some college 27.3%; College graduate 0.5%; Post graduate 3.2%  *Employment:* Employed full time 28.7%; Employed part time 29.8%; Homemaker 9.0%; Retired 1.1%; On disability 1.1%; Student 0.5%; Unemployed 27.6%; Other 2.1%  *Income:* Mean monthly family income 698.0 (SD=487.4)  *Family size:* Mean 2.8 (SD=1.5)  *Spoken language:* Spanish only 17.5%; Spanish spoken as a child 96.8%  *Spanish as preferred language:* Read 95.2%; Speak at home 94.1%; Speak with friends 92.6%; Think 93.6%; Watch television 83.5%; Listen to the radio 88.2% | FIT Uptake   - 90% of participants complied with CRC screening using the FIT. - 87% of the participants returned their FIT within 2 weeks. - Almost 70% of participants completed FIT without any telephone reminders. - 19.3% of participants returned their FIT after a single reminder.   Facilitators   - The intervention targeted low-income Latinas who lacked medical insurance; thus, it removed financial and access to care barriers. - The intervention addressed socio-cultural barriers as bilingual lay health educators were linguistically and culturally related to the target population. | Only focused on low-income, uninsured Latinas which are a specific underserved population; use of acculturation measures that do not account for multidimensional factors affecting non-linear adaptation within immigrant populations; and requirement of all participants to receive the single intervention with an absence of a comparison group.  Moderate |
| Hillyer et al., 2014  United States  Compared the impact of gFOBT and FIT programs for uninsured, minority, and low socioeconomic individuals over a 13-year period. | Retrospective record review (retrospective record review)  Participants were recruited through health fairs, political liaisons, local businesses, senior centers, schools, churches, faith-based organizations, housing projects, social and cultural groups, and social clubs. Bilingual lay health educators recruited participants.  Lay educators spoke with participants at the time of enrollment about colorectal cancer, risk factors, and the importance of early detection. Patients were provided a gFOBT or a FIT.  Tailoring   - The intervention was delivered either in English or Spanish based on participants preference. - Materials were prepared at a sixth-grade reading level. | *Population size:* 7,710 participants (n=4,951 gFOBT; n=2,759 FIT)  *Age:* 50-54 53.9%; 55-59 25.5%; 60-64 13.7%; ≥65 6.9%  *Sex:* Female 85.4%; Male 14.6%  *Race:* Asian 26.0%; Black/African American 16.1%; White 44.1%; Other 13.8%  *Ethnicity:* Hispanic 51.5%^^[[3]](#footnote-3)^^  *Nativity:* Foreign born 86.1%  *Note:* Population demographics reported for participants in the FIT intervention only. | FIT Uptake   - 2,759 participants completed the FIT. - Those using FIT were significantly younger (50-54) (p<0.001), females (p<0.001), white (p<0.001), and Hispanic (p<0.001) versus those using gFOBT. - Participants returned FIT faster than gFOBT (23.3 days versus 26.5 days p<0.001).   Facilitators   - Patient navigation supported minority and disadvantaged populations. | Limited generalizability due to study design; and unaccounted confounding variables reflected in statistically significant differences in demographic population.  Weak |
| Hirko et al. 2020  United States  Analyzed whether motivational messaging combined with FIT increases colorectal cancer (CRC) screening in a rural setting. | Randomized controlled trial (randomized controlled trial)  Asymptomatic individuals between the ages of 50-75 were identified using the MidMichigan Health data registry.  The intervention contained a mailed motivational outreach letter. It was given the option to participants to call and request a free FIT. The control group received the usual care mailed letter.  Tailoring   - The intervention group’s mailed letter was specifically designed to address identified barriers in screening among the rural population. - Motivational messaging used six core motivators (i.e., support, testimony, empowerment, control, physical survivor/expectation, trust, options, and affordability) for educating, empowering, and mobilizing those who have not attended CRC screening. | *Population size:* 7,812 participants (n=3,906 usual care control group; n=3,906 intervention group)  *Sex (Usual care):* Female 53.2%; Male 46.8%  *Sex (Intervention):* Female 54.1%; Male 45.9%  *BMI category (Usual care):* Normal 20.6%; Overweight 27.7%; Obese 51.8%  *BMI category (Intervention):* Normal 18.7%; Overweight 27.8%; Obese 53.5%  *Previous colorectal cancer screening (Usual care):* Yes 21.0%; No 79.0%  *Previous colorectal cancer screening (Intervention):* Yes 20.6%; No 79.4% | FIT uptake   - 233 (6%) participants in the intervention group requested a FIT. - Of these 233 participants, 154 (66%) returned the FIT. - Overall, there was a 7.6% increase in CRC screening in the intervention group compared to the control group. - No significant difference in FIT request, return, and follow-up was found between men and women. | Lack of socioeconomic and demographic information; inability to assess potential differences in screening test modality; and unavailability of residential information not allowing assessment of differences in degree of rurality.  Weak |
| Lee et al., 2020  United States  Evaluated the effectiveness of a phone outreach to increase the subsequent completion rate of mailed-out FIT among populations with prior FIT completion. | Randomized controlled trial (controlled clinical trial)  Participants were sampled from the San Francisco Health Network, which is a publicly funded safety-net health system serving low-income populations.  Participants who had previous negative FIT testing were randomized to receive either the advanced notification phone call (advanced call group) or no advanced phone notification call (no advanced call group). The advance call group received two advance notification phone calls prior to the mail-out delivery of FIT. The no advance phone call groups were only called once prior to the mail-out delivery of FIT. Both groups received reminder phone calls after delivery of the mail-out FIT if not returned in two weeks. | *Population size:* 3,240 participants (n=1,595 “no advanced call” group; n=1,645 “advanced call” group)  *Age (No call):* 50-54 17.9%; 55-59 28.8%; 60-64 25.6%; 65-69 19.0%; 70-75 8.8%  *Age (Call):* 50-54 18.4%; 55-59 26.3%; 60-64 27.7%; 65-69 18.0%; 70-75 9.7%  *Sex (No call):* Female 50.7%; Male 49.3%  *Sex (Call):* Female 50.3%; Male 49.7%  *Ethnicity (No call):* Hispanic 25.6%; Non-Hispanic Black 19.2%; Non-Hispanic White 19.1%; Asian 23.2%; Other/unknown 12.9%  *Ethnicity (Call):* Hispanic 24.8%; Non-Hispanic Black 18.9%; Non-Hispanic White 18.8%; Asian 23.7%; Other/unknown 13.7%  *Marital status (No call):* Single 48.6%; Married 28.0%; Divorced 9.5%; Separated 4.9%; Widowed 4.8%; Unknown 4.2%  *Marital status (Call):* Single 48.8%; Married 26.1%; Divorced 9.2%; Separated 5.7%; Widowed 5.2%; Unknown 5.0%  *Insurance (No call):* Medicaid 47.5%; Medicare 18.4%; County-sponsored 10.4%; Healthy worker 17.3%; Uninsured 4.6%; Other/unknown 1.8%  *Insurance (Call):* Medicaid 48.7%; Medicare 20.1%; County-sponsored 8.0%; Healthy worker 16.3%; Uninsured 5.1%; Other/unknown 1.9%  *Primary language (No call):* English 58.7%; Spanish 22.3%; Chinese 9.0%; Other/unknown 10.0%  *Primary language (Call):* English 57.1%; Spanish 22.1%; Chinese 9.8%; Other/unknown 10.9% | FIT uptake   - There were no significant differences in advance call group and no advance call group completion rate of FIT after one year (70.9% and 69.9%, respectively). - Of the participants in the advance call group who were spoken to or received a voicemail (90.5%) were significantly more likely to complete FIT within one year than those who were only left a voicemail or could not be reached (p<0.01). | Loss of follow-up of participants impacting analysis; unaccounted confounding variables; lack of generalizability as study was conducted in a singular safety-net health system; and not having the capacity to use an advanced notification system that utilized postcards, automated calls, SMS, or other forms of electronic communication.  Moderate |
| Lee et al., 2022  United States  Assessed the effectiveness over time of outreach methods to increase subsequent completion rate of mailed-out FIT among populations who were not up-to-date with their colorectal cancer (CRC) screening. | Pragmatic trial (controlled clinical trial)  Participants were sampled from the San Francisco Health Network, which is a publicly funded safety-net health system serving low-income populations.  Participants who had previous negative colorectal cancer testing were randomized to either the outreach group or the usual care group. The outreach group received advance notification postcards and phone calls prior to the mail-out delivery of FIT and up to two reminder phone calls if FIT was not returned after two weeks. The usual care group followed directions according to providers in the participating clinics which may have included reminders, tailoring of education materials, coaching and outpatient visits.  Tailoring   - Phone calls had available language and interpretive services for participants. | *Population size:* 10,771 participants (n=5,410 usual care; n=5,361 outreach)  *Age (Usual care):* 50-54 27.0%; 55-59 26.7%; 60-64 23.3%; 65-69 15.4%; 70-74 7.7%  *Age (Outreach):* 50-54 26.2%; 55-59 26.5%; 60-64 23.4%; 65-69 15.9%; 70-74 7.9%  *Sex (Usual care):* Female 47.5%; Male 52.5%  *Sex (Outreach):* Female 46.8%; Male 53.2%  *Ethnicity (Usual care):* Hispanic 25.1%; Non-Hispanic Black 22.3%; Non-Hispanic White 22.6%; Asian 19.2%; Other/unknown 10.9%  *Ethnicity (Outreach):* Hispanic 24.2%; Non-Hispanic Black 22.1%; Non-Hispanic White 23.0%; Asian 19.3%; Other/unknown 11.4%  *Marital status (Usual care):* Single 48.6%; Married 21.3%; Divorced 8.0%; Separated 4.4%; Widowed 4.0%; Unknown 13.7%  *Marital status (Usual Outreach):* Single 48.6%; Married 20.9%; Divorced 8.4%; Separated 4.4%; Widowed 3.9%; Unknown 13.7%  *Insurance (Usual care):* Medicaid 50.2%; Medicare 19.2%; County-sponsored 8.8%; Healthy worker 13.4%; Uninsured 5.1%  *Insurance (Outreach):* Medicaid 50.8%; Medicare 18.5%; County-sponsored 8.5%; Healthy worker 14.1%; Uninsured 5.1%  *Primary language (Usual care):* English 65.7%; Spanish 19.2%; Chinese 5.7%; Other/unknown 11.1%  *Primary language (Outreach):* English 65.0%; Spanish 19.8%; Chinese 6.9%; Other/unknown 8.3% | FIT uptake   - There was significantly more uptake of FIT in the outreach group than the usual care group after two and a half years (73.2% versus 55.1%, p<0.001). - In participants who had completed a previous FIT test, there was a significant increase in FIT completion than among participants who had no prior FIT test completion (83.9% versus 71.8% p<0.001). - Participants in the outreach group were significantly less likely to be nonadherent (0/2 expected FIT) than participants in the usual care group (26.8% versus 44.9%, p<0.001). - The proportion of time up-to-date with FIT screening was 46.8% in the outreach group and 27.3% in the usual care group (difference of 19.6%; 95% CI: 18.2%–20.9%). | Lack of generalizability as study was conducted in a singular safety-net health system; used annual stool-based screening versus biannual stool-based screening guidelines for analysis; and loss of follow-up of participants impacting analysis.  Weak |
| Lucas et al., 2021  United States  Examined the impacts of a standard and culturally targeted messaging using a loss-framing and gain-framing approach on receptivity, behaviour (receiving and returning FIT), and anticipatory racism among African Americans. | Fully-crossed between- participants experimental design (comparative study)  Participants were recruited through face-to-face outreach and online advertisement in the metropolitan Detroit community area.  Participants watched an online information video module about colorectal cancer screening. Participants were randomly assigned in the videos to either receive gain-framed (benefits of getting screening done) or loss-framed messages (costs, losses, and risks of not getting screening done). Half of participants were then randomly assigned to receive an additional culturally targeted personal prevention messaging. At the end, participants were offered to receive a FIT through the mail at no cost, which would also be returned by mail after completion.  Tailoring   - Videos were culturally developed for African Americans. - Cost-free FIT. | *Population size:* 457 participants  *Age:* 50-60 67.4%; 61-70 29.2%; 71 & above 3.4%  *Sex:* Female 74.4%; Male 25.6%  *Education:* High school/GED 27.6%; Some colleague or trade school 46.6%; College graduate 16.4%; Professional/advanced degree 9.4%  *Insurance:* Medicare/Medicaid 49.9%; Private 31.7%; Combination of government/private 9.0%; No insurance 9.4%  *Income:* Less than $25,000 41.6%; $25,000-$49,999 30.4%; $50,000-$99,999 21.7%; $100,000 and above 6.3% | FIT uptake   - Overall, 292 (63.9%) participants asked to receive FIT, of which only 56 participants returned FIT (19.18%). - There were no significant differences in receiving and in returning the FIT between the groups. - Participants in the culturally targeted condition were more receptive to CRC screening, compared to the standard condition. - Culturally targeted loss-framing had significantly more CRC receptivity than participating receiving standard loss-framing messaging (p<0.005, d=0.374) - Participants were least receptive to standard loss-framed messaging and more receptive to standard gain-framed or culturally targeted loss-framed messaging, but overall, none of these groups were found to be statistically significant. - While targeted loss-framing reduced anticipation of racism, the standard loss-framing increased it. Culturally targeted loss-framed messaging was found to lower anticipatory racism (p<0.05, d=2.99).   Barriers   - Standard loss-framed messaging did not improve African American CRC screening because messages may be triggering racism-related emotions, thus reducing the message's persuasiveness. | Not reaching intended effect size impact because the sample size calculations were not met; limited reproducibility into a large-scale program to reduce racial disparities; limited generalizability to African Americans; inability to track use of colonoscopy and sigmoidoscopy; and messaging limited to group-level characteristics and not combined with a tailored messaging approach.  Weak |
| Prakash et al., 2022  United States  Retrospectively examined whether a colorectal cancer (CRC) screening program (named GET FIT) utilizing a telephone and letter reminder system increased the FIT return rate. | Retrospective quality improvement study (randomized study with no control group)  Participants were enrolled in the program at public outreach events, and at daily internal and external outpatient clinics.  Participants in the GET FIT program were given education on CRC and FIT as a method of screening. They also received a FIT with instructions on how to perform the test and how to mail the completed test back. Participants who did not return the FIT within two weeks were further randomized into 1) telephone reminders or 2) telephone reminders and letters biweekly over a period of 60 days or five attempts of contact. | *Population size:* 959 participants (n=460 *telephone reminder*; n=499 *telephone reminder and letter*)  *Sex (telephone reminder):* Female 66.9%; Male 32.6%  *Sex (telephone and letter reminder):* Female 61.9%; Male 38.1%  *Ethnicity (telephone reminder):* White 43.5%; Hispanic 48.5%; Black 4.6%; Other 3.4%  *Ethnicity (telephone and letter reminder):* White 39.9%; Hispanic 51.1%; Black 3.4%; Other 5.6% | FIT uptake   - 283 of 460 (61.5%) participants returned the FIT. - 247 of 279 (88.3%) participants returned the FIT without receiving any reminders - 81 of 106 (76.4%) participants returned the FIT after receiving phone call reminders. Within this group, 79% of participants returned after one call, 13.6% after two calls, and 7.4% after three or more calls. - 27.2% of participants who received both phone and letter reminders returned the FIT. - There was no significant difference in FIT return rate between females and males (p=0.36). - There was a significant difference in FIT return rate between races/ethnicities. Individuals who were Black (82.3%) or Hispanic (77.25%) had the highest return rates. | Lack of participants who only received the reminder letter; and low sample size to evaluate the impact of the reminder methods by ethnicity.  Weak |
| Ritzenthaler et al., 2022  United States  Investigated whether the live telephone outreach intervention increased rates of colorectal cancer (CRC) screening. | Prospective, single-arm cohort study (prospective, single-arm cohort study)  Patients in a community health centre located in a medically-underserved neighbourhood were called. There were three outcomes based on the call: 1) patient interaction with motivational interviewing, 2) voicemail with CRC screening reminders, and 3) terminal outcomes such as refusal, FIT completion, or invalid number.  Based on the recruitment outcome, patients were mailed FIT and given live reminders if they were not returned.  Tailoring   - Telehealth outreach and reminders were used to address disruptions to CRC screening during the COVID-19 pandemic. | *Population size:* 643 participants  *Age:* Median 61 years old  *Sex:* Women 59.2%  *Race:* Black 91.7%; Non-Hispanic White 5.0%  *Language:* English-speaking 99.8%^^[[4]](#footnote-4)^^ | FIT uptake   - Overall, the FIT completion rate was 28.4% among the 338 patients who had at least one FIT ordered, which was significantly higher than patients who did have any ordered during their patient interaction. - 123 participants (85.4%) who completed FIT had at least one interaction with the outreach team. - 47 participants (32.6%) had at least one voicemail. - 142 participants (98.6%) had at least one successful contact. - Patients who had at least one interaction or successful contact had significantly higher FIT completion rates compared to no contact (p<0.0001). - FIT completion rates were significantly lower among patients without valid phone numbers (4.1%; P<0.01) or voicemails (11.8%; p<0.001).   Barriers   - Patients faced technology-based barriers, such as lack of access to a working phone or voicemail. These barriers were significantly associated with lower FIT uptake. | Lack of control arm; telephone outreach not suitable for younger populations; continuously evolving telecommunications technology; lack of generalizability because the included population was predominantly Black (90%); and COVID-19 pandemic causing difficulties in measuring the effectiveness of the intervention.  Weak |
| Sali et al., 2016  Italy  Evaluated the effectiveness of different colorectal cancer screening (CRC) methods to improve participation rates. | Randomized controlled trial (randomized controlled trial)  Participants were recruited from municipal population files in Florence, Italy.  Participants were randomly assigned to be invited to either: biennial FIT for three rounds, single r-CTC (reduced computed tomography colonography (CTC)), single f-CTC (full cathartic preparation CTC), or optical colonoscopy (OC). | *Population size:* 16,087 participants (n=9,739 invited to FIT, n=9,288 eligible; n=2,617 invited to reduced cathartic preparation CT colonography, n=2,935 eligible; n=2,625 invited to full cathartic preparation CT colonography, n=2,430 eligible; n=1,106 invited to optical colonoscopy, n=1,036 eligible)  *Age:* 54-60 58.0%; 61-65 42.0%  *Sex:* Female 56.4%; Male 43.6%  *Socio-economic status:* Low 43.2%; Average 28.1%; High 25.5%^^[[5]](#footnote-5)^^  *Note:* Population demographics reported for participants in the FIT intervention only. | FIT Uptake   - 50.4% of invitees participated in the first round of FIT. - FIT had significantly higher participation rates (p<0.001 for all groups). - Compared to all the groups, the FIT group had significantly higher participation of women (p=0.002, p<0.001, p=0.002) and individuals over the age of 60 (p=0.02, p=0.007, p=0.03). - FIT participants of low SES had lower participation compared to the ones of high SES. | Only analyzed results after one round of biennial FIT; and underpowered sample size.  Weak |
| Sali et al., 2018  Italy  Evaluated the participation in FIT screening among non-attendees for computed tomography colonography (CTC) and optical colonoscopy (OC). | Randomized controlled trial (randomized trial with no control group)  Participants were recruited from a district in Florence, Italy.  Participants were randomly assigned to be invited to either biennial FIT, reduced CTC, full CTC, or OC group. Non-attendees of all groups were reminded through the mail and invited to FIT. | *Population size:* 964 participants (n=347 reduced-preparation CTC non-attendees but completed FIT; n=389 full-preparation CTC non-attendees but completed FIT; n=228 OC non-attendees but completed FIT)  *Age:* 54-60 55.7%; 61-65 44.3%  *Sex:* Female 63.0%; Male 37.0%  *SES:* Low 42.4%; Medium 28.1%; High 24.8%^^[[6]](#footnote-6)^^ | Fit Uptake   - Among the non-attendees in reduced CTC, full CTC, and OC that participated in reminder mail-out FIT, participation was 20.2%, 21.4%, and 25.8% respectively. - Overall, participation rate for FIT was higher among individuals older than 60 years of age (p<0.05) and women (p<0.005). | Results are based only on a single round of FIT.  Weak |
| Somsouk et al., 2020  United States  Evaluated the effectiveness, costs, and cost-effectiveness of an outreach intervention to improve colorectal cancer (CRC) screening. | Randomized controlled trial (controlled clinical trial)  Participants were sampled from the San Francisco Health Network, which is a publicly funded safety-net health system serving low-income populations.  Participants who were not up-to-date with their CRC screening (FIT, sigmoidoscopy, colonoscopy) were randomly assigned to either the outreach group or usual care group. The outreach group received an informational postcard and two phone calls prior to the mail-out delivery of FIT and up to two reminder phone calls upon the non-return of FIT within two weeks. The usual care group followed directions according to discretion of healthcare providers in the participating clinics and those participants were offered FIT during the visit.  Tailoring   - In the outreach group, the FIT contained low-literacy instructions (with no words) on how to complete the test. - In the outreach group, the FIT materials were available in English, Spanish, and Chinese. | *Population size:* 10,820 participants (n=5,434 usual care arm; n=5,386 intervention arm)  *Age (Usual care):* 50-55 27.1%; 55-60 26.6%; 60-65 23.3%; 65-70 15.3%; 70-75 7.7%  *Age (Intervention):* 50-55 26.2%; 55-60 26.6%; 60-65 23.4%; 65-70 15.8%; 70-75 8.0%  *Sex (Usual care):* Female 47.5%; Male 52.5%  *Sex (Intervention):* Female 46.8%; Male 43.2%  *Ethnicity (Usual care):* Hispanic 24.2%; Non-Hispanic Black 21.7%; Non-Hispanic White 21.8%; Asian 18.8%  *Ethnicity (Intervention):* Hispanic 23.5%; Non-Hispanic Black 21.8%; Non-Hispanic White 22.4%; Asian 18.8%  *Insurance (Usual care):* Medicaid 49.8%; Medicare 18.9%; Uninsured 5.2%; County sponsored 8.6%; Healthy worker 13.4%  *Insurance (Intervention):* Medicaid 50.3%; Medicare 18.2%; Uninsured 5.1%; County sponsored 8.3%; Healthy worker 14.1%  *Language (Usual care):* Chinese 4.3%; Spanish 12.4%; English 48.5%; Other/unknown 34.8%  *Language (Intervention):* Chinese 6.8%; Spanish 19.4%; English 64.3%; Other/unknown 9.5% | FIT uptake   - After one year, 57.9% of participants in the outreach group returned FIT, whereas only 37.4% of participants in the usual care group completed FIT (difference of 20.5%; 95% CI: 18.6%-22.4%; p<0.001). - After 28 days, there were significantly more FIT completed in the outreach group versus the usual care group (13.3% versus 5.1%, p<0.001). - Subgroup analysis showed that the highest screening participation at one year was among ethnic minorities (69.3% of Asians and 65.2% of Hispanics), and patients who used non-English languages (80% Chinese and 68.2% Spanish). - Participants with no prior FIT were significantly more likely to complete a FIT than those with prior FIT (70.2% versus 34.8%, p<0.001).   Referral   - After six months of receiving an abnormal FIT, only 51.0% and 51.4% of outreach and usual care group participants, respectively, followed-up for colonoscopy.   Barriers   - After reminder phone calls, participants cited forgetting/being busy (62.7%), lost or damaged FIT (62.7%), and not understanding how to complete the test (77.4%) as the main reasons for not returning the FIT. | Sampling bias of clinics that allowed for patient recruitment; exclusion of patients who were homeless and had comorbidities; and no subgrouping or stratification or analysis of each step of the intervention to determine effectiveness attribute to which component.  Weak |
| Stone et al., 2019  United States  Investigated the effectiveness of the colorectal cancer (CRC) screening using home visits and phone calls among age-eligible African Americans living in public housing units or relying on housing subsidies. | Not specified (cohort one group)  Patients were recruited through phone calls and home visits using the Louisville Metropolitan Housing Authority (LMHA). Lastly, three mailings were delivered at 6-week intervals to recruit participants into the study.  Paper surveys were collected during face-to-face interviews with participants who also received FIT and a $25 gift card. The interviewers educated the participants on how to collect FIT sample and how to mail-out the completed FIT. | *Population size:* 209 participants  *Sex:* Female 85.6%  *Ethnicity:* African American 100%  *Marital status:* Married 13.4%; Single 67.5%  *Education:* Less than high school education 33.5%  *Insurance:* Insured 96.7%  *Regular provider:* Yes 95.2%  *Income:* Income below $10,000 82.8%  *Large public housing development resident:* Yes 69.9%  *BMI:* BMI calculated (>25) 80.4%; BMI perceived (>25) 45.1%  *Prior CRC screen:* Yes 52.2%  *Family history:* Cancer 55.0%; CRC 7.2%  *Smoker:* Yes 48.8%  *Alcohol consumption:* No alcohol in last 30 days 26.3%  *Exercise:* Exercise in past week 62.2%  *Perceived health:* Fair/poor 41.6% | FIT uptake   - 149 of 209 participants (71.3%) returned the FIT. - Residents of large public housing were 1.87 times (95% CI: 0.987-3.552; p=0.055) more likely to return the FIT compared to participants living in scattered housing or rental units. - Participants who self-rated their health as fair or poor were two times (95% CI: 1.053-3.821; p =0.034) more likely to return the FIT. - The home visits may be more effective in increasing CRC screening compared to mailing FIT. | Lack of representation of minority groups due to small sample size; difficulty to generalize the study; limited predictors related to FIT uptake; and unintended impact of financial incentive in participation.  Moderate |
| Symonds et al., 2019  Australia  Examined whether offering a blood screening test (upfront choice or rescue) within a mail-based FIT-based screening program would improve colorectal cancer screening (CRC) among participants. | Randomized controlled trial (randomized controlled trial)  All individuals in the regions of South Australia were mailed-out invitations to participate in CRC screening. Individuals were provided with a phone number to opt out of the screening.  Participants who did not opt-out of screening were randomized into the control, choice, or rescue group. The control group was just sent a FIT kit with reminders of FIT completion after 12 weeks and 18 weeks. The choice group was sent a FIT or could complete a blood test instead and were not sent reminders to complete CRC. The rescue group followed the same process as the control group, but the 12-week and 18-week reminders offered a choice of a FIT or a blood test.  Tailoring   - Reply-paid return envelopes for sending the sample tubes of the FIT to the laboratory. | *Population size:* 1,800 participants (n=600 control; n=600 rescue; n=600 choice)  *Age (control):* 50-55 20.3%; 56-60 18.3%; 61-65 19.3%; 66-70 21.6%; 71-74 20.3%  *Age (rescue):* 50-55 20.5%; 56-60 22.5%; 61-65 17.0%; 66-70 20.3%; 71-74 19.6%  *Age (choice):* 50-55 19.1%; 56-60 19.1%; 61-65 23.6%; 66-70 18.0%; 71-74 20.0%  *Sex (control):* Females 48.5%; Males 51.5%  *Sex (rescue):* Females 53.0%; Males 47.0%  *Sex (choice):* Females 48.5%; Males 51.5%  *Relative socioeconomic disadvantage (control):* Most disadvantaged 35.3%; Least disadvantaged 64.6%  *Relative socioeconomic disadvantage (rescue):* Most disadvantaged 40.6%; Least disadvantaged 59.3%  *Relative socioeconomic disadvantage (choice):* Most disadvantaged 37.6%; Least disadvantaged 62.3% | FIT Uptake   - No significant difference in participation at 12 weeks and 24 weeks was found between control, rescue, and choice group. - Re-invitation strategies at 12 and 18 weeks in the control and rescue group did not significantly increase participation in CRC (2.6% FIT uptake and 5.3% FIT or blood test).   Barriers   - Fecal aversion may be impacting participation uptake. - The need to go to a medical center for the blood test acted as a barrier to participation. | Blood test group required more information which may have led to information overload and confusion among participants; data collection may have biased participants; and unequal reminder letters sent between groups (control and rescue versus choice).  Weak |
| Turrin et al., 2015  Italy  Compared the Italian colorectal cancer (CRC) screening programmes in immigrants born in low- or middle-income countries and native populations. | Not specified (cross-sectional study)  A national survey was distributed to collect information on the CRC screening programmes. Within the survey information was collected on immigrants.  No intervention. | *Population size:* 3,099,822 invited participants (n=1,556,647 Italians screened; n= 58,612 immigrants screened)  *Age (Italians):* 50-59 50.8%; 60-69 49.2%  *Age (Immigrants):* 50-59 74.2%; 60-69 25.8%  *Sex (Italians):* Female 53.7%; Male 46.4%  *Sex (Immigrants):* Female 66.1%; Male 33.9% | FIT uptake   - Participation was significantly lower among immigrants compared to native Italians (p<0.001). - In both groups, screening rates were higher among women compared to men. - Compliance to screen after already being screened was higher in native Italians compared to immigrants (p<0.001). - Immigrants who were invited for the first time to screen were significantly less likely to comply compared to native Italians (p<0.001), but there was no significant difference among those who previously refused screening (p=0.03). - Among all groups (i.e., immigrant women, immigrant men, native Italian women, native Italian men), immigrant males had the lowest compliance rates. - Compliance rates were higher among immigrant women in the younger age group and among native Italian women in the older age group.   Referral   - There were no differences in compliance with colonoscopy after a positive FIT between immigrant and native groups. | Voluntary participation of the immigrant population in the national survey; and none of the screening programmes in certain regions in Italy provided their data; and lack of country and area specific data.  Weak |
| van der Meulen et al., 2022  Netherlands  Examined the socioeconomic differences in participation in the national Dutch FIT screening program. | Not specified (ecological study)  Data from the study came from the Dutch national screening program with biennial FIT data.  No intervention; only an analysis of data from the Dutch National screening program. Researchers utilized socioeconomic data (income, employment status, and education level) to stratify residents into quintiles from highest to lowest socioeconomic (SES) to make comparisons (quintile 1 to quintile 5). | *Population size:* 1,866,060 invited participants with an area-based SES score (n=1,360,103 attendance to FIT)  *Age:* Median 65.9 years  *Sex:* Male 49.3%  *SES:* Quintile 1 18.1%; Quintile 2 21.1%; Quintile 3 22.3%; Quintile 4 20.9%; Quintile 5 17.6%  *Note:* SES reported for attendance to FIT participants only, not the invited sample. | FIT Uptake   - Using Quintile 1 (73.9% participation) as reference, it was found Quintile 2 (75.1%; OR=1.07; 95% CI: 1.06-1.08) and 3 (75.1%; OR=1.07; 95% CI: 1.06-1.08) had more FIT uptake. The uptake was lower in Quintile 4 (73.0%; OR=0.96; 95% CI: 0.95-0.97) and Quintile 5 (67.0%; OR=0.73; 95% CI: 0.72-0.74).   Referral   - There was significantly lower participation in colonoscopy after a positive FIT for individuals with low SES (Quintile 4: OR=0.93; 95% CI: 0.88-0.98; and Quintile 5: OR=0.73; 95% CI: 0.69-0.77) than individuals with high SES.   Barriers   - The Dutch health system provides an obligatory co-payment for delivered care during a calendar year. Participants may have postponed procedures if the co-payment is maximized within the year, thus delaying or foregoing colonoscopy. | Use of aggregated data on SES with inaccurate representation of SES at the individual level (ecological fallacy).  Moderate |
| Young et al., 2021  Australia  Examined different strategies in engaging non-participants from a previous study to improve colorectal cancer screening. | Randomized controlled trial (randomized trial with no control group)  Participants in this study were recruited from a previous study, in which participants identified through an electoral list received a mailed invitation but failed to complete colorectal cancer screening.  Participants were randomized to either receive a mailed invitation to FIT, arrange for a blood test, or the choice to do either FIT or the blood test. Reminders to complete testing were sent out at week six and 12 weeks.  Tailoring   - Prepaid return envelopes for the FIT were provided. | *Population size:* 875 participants invited (n=292 invited to FIT, n=35 participated; n=293 invited to blood test, n= 39 participated; n=290 invited to choice, n=39 participated); overall n=63 participants participated by FIT  *Age:* 50-55 12.7%; 56-60 23.8%; 61-65 27.0%; 66-70 12.7%; 71-74 23.8%  *Sex:* Female 46.0%; Male 54.0%  *Relative socioeconomic disadvantage:* Most disadvantaged 23.8%; Least disadvantaged 76.2%  *Note:* Population demographics reported for participants in the FIT intervention only. | FIT Uptake   - No significant difference in participation were found between FIT, blood test, and or choice group. - Within the choice group, significantly more participants chose FIT (9.7%) than blood tests (3.8%; p=0.005). - Among FIT participants, relative socioeconomic disadvantage was associated with participation. - In the choice group, participants who selected FIT indicated familiarity, convenience, comfort, and time-saving as factors contributing to their decision.   Barriers   - Among blood test participants, fecal aversion was a reason in their choice of screening strategy. - Blood tests being conducted at a medical center may be a barrier to participation. | Generalizability limited to mail-out FIT; novelty of blood test for CRC screening; and low survey response resulting in low power of findings.  Weak |

^a^ The study design is presented as reported by authors’ papers. In parenthesis is the articulation of the study design which aligns with the parameters from the Effective Public Healthcare Panacea Project (EPHPP) *Quality Assessment Tool for Quantitative Studies* {Effective Public Healthcare Panacea Project, n.d. #1031}.

1. UD1 = very high urban density (≥2500 addresses/km^2^); UD2 = high urban density (1500-2500 addresses/km^2^); UD3 = intermediate urban density (1000-1500 addresses/km^2^); UD4 = low urban density (500-1000 addresses/km^2^); UD5 = very low urban density (≤500 addresses/km^2^). [↑](#footnote-ref-1)
2. SES1 = very high SES; SES5 = very low SES [↑](#footnote-ref-2)
3. Other ethnicities were not reported. [↑](#footnote-ref-3)
4. Note: Population demographics are reported for the patients (n=824) who were due for colorectal cancer screening; only 643 met inclusion criteria; specific demographics for the subset of the participants that met inclusion criteria was not reported. [↑](#footnote-ref-4)
5. Missing values for SES. [↑](#footnote-ref-5)
6. Missing values for SES. [↑](#footnote-ref-6)
